# A comparative characterisation of commercially available lipid-polymer nanoparticles formed from model membranes

**DOI:** 10.1007/s00249-023-01632-5

**Published:** 2023-02-14

**Authors:** Henry Sawczyc, Sabine Heit, Anthony Watts

**Affiliations:** Department of Biochemistry, University of Oxford, South Parks Road, Oxford, OX1 3QU UK

**Keywords:** Lipid-polymer nanoparticles, SMALPs, Lipodisqs, DIBMA, PMA, Nanodiscs

## Abstract

From the discovery of the first membrane-interacting polymer, styrene maleic-acid (SMA), there has been a rapid development of membrane solubilising polymers. These new polymers can solubilise membranes under a wide range of conditions and produce varied sizes of nanoparticles, yet there has been a lack of broad comparison between the common polymer types and solubilising conditions. Here, we present a comparative study on the three most common commercial polymers: SMA 3:1, SMA 2:1, and DIBMA. Additionally, this work presents, for the first time, a comparative characterisation of polymethacrylate copolymer (PMA). Absorbance and dynamic light scattering measurements were used to evaluate solubilisation across key buffer conditions in a simple, adaptable assay format that looked at pH, salinity, and divalent cation concentration. Lipid-polymer nanoparticles formed from SMA variants were found to be the most susceptible to buffer effects, with nanoparticles from either zwitterionic DMPC or POPC:POPG (3:1) bilayers only forming in low to moderate salinity (< 600 mM NaCl) and above pH 6. DIBMA-lipid nanoparticles could be formed above a pH of 5 and were stable in up to 4 M NaCl. Similarly, PMA-lipid nanoparticles were stable in all NaCl concentrations tested (up to 4 M) and a broad pH range (3–10). However, for both DIBMA and PMA nanoparticles there is a severe penalty observed for bilayer solubilisation in non-optimal conditions or when using a charged membrane. Additionally, lipid fluidity of the DMPC-polymer nanoparticles was analysed through cw-EPR, showing no cooperative gel-fluid transition as would be expected for native-like lipid membranes.

**Supplementary Information:**

The online version contains supplementary material available at 10.1007/s00249-023-01632-5.

## Introduction

Membrane proteins account for over 60% of all drug targets (Overington and Bissan Al-Lazikani 2006), while comprising only approximately 25% of the genome (Bakheet and Doig 2009). Despite the therapeutic interest in membrane proteins, these proteins account for only ~ 2% of depositions in the Protein Data Bank (PDB) (Berman et al. 2000; Newport et al. 2019). The lack of structures can be partially attributed to the challenge of successfully solubilising membrane proteins for characterisation, in addition to the difficulties in overexpression of these systems (Carpenter et al. 2008; Zorman et al. 2015).

**Fig. 1:**
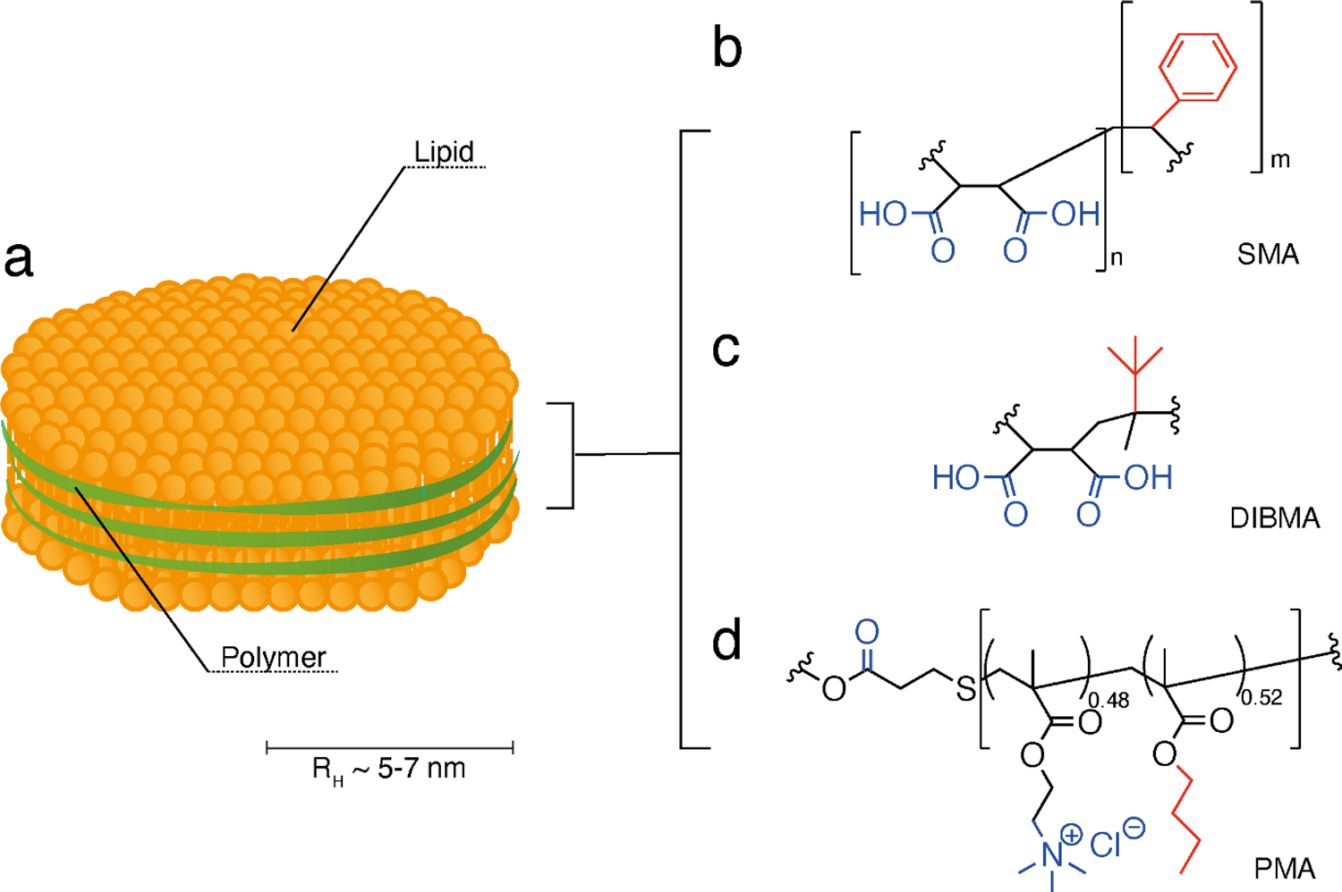
Diagram of polymer structure used for forming polymer-lipid nanoparticles. **a** Cartoon representation of polymer-lipid nanoparticles resulting from interaction with polymers (**b**–**d**), comprised of lipids (shown in yellow) surrounded by a polymer ‘belt’ (shown in green). Multiple polymers have been shown to be capable of forming nanoparticles, such as: **b** SMA, **c** DIBMA, and **d** PMA. The hydrophilic groups are highlighted in blue, and hydrophobic groups highlighted in red

Multiple techniques have been developed to aid in membrane protein solubilisation, with the most common method utilising detergents. Detergents solubilise membrane proteins through disruption of the membrane bilayer which leads to discrete micelles of lipid, detergent, and membrane protein (Seddon et al. 2004). Numerous detergent-based solubilisation methods have been developed, and are widely used for not only membrane protein solubilisation, but also as a model system for membrane protein characterisation, and structural determination (Skrzypek et al. 2018; Choy et al. 2021). However, detergent-based methods have drawbacks, including the increased possibility of protein denaturation (Tulumello and Deber 2012; Lee et al. 2016), and loss of native protein-lipid interactions through delipidation (Ilgü et al. 2014; Champeil et al. 2016). The addition of detergents can also lead to the disassembly of membrane complexes (Gupta et al. 2017). The negative aspects of detergents have led to the development of several model membrane systems such as vesicles, peptide nanodiscs, or amphipols (Tribet et al. 1996; Denisov et al. 2004; Borch and Hamann 2009; Zoonens and Popot 2014). These systems are *in lieu* of detergent micelles during protein characterisation, but still require the presence of detergent for the solubilisation and purification process. A viable solution to detergent-based purification came in the form of discoidal polymer-based nanoparticles, in which integral membrane proteins can be incorporated to form lipid-polymer nanoparticles (Fig. 1a). The formation of these lipid-polymer nanoparticles can be achieved without the need for detergent at any step and allows for retention of native lipids when extracting membrane proteins (Smirnova et al. 2018; Bada Juarez et al. 2021). Within the native lipid environment, the membrane protein of interest often possesses increased stability and more native-like activity compared to detergent-based purifications (Triano et al. 2010; Orwick-Rydmark et al. 2012; Bada Juarez et al. 2020).

The first lipid-polymer nanoparticles were generated using styrene maleic-acid (SMA), in 2006 (Tonge 2006; Bada Juarez et al. 2019). SMA is an amphipathic polymer that contains similar functional groups to those seen in detergents. The functional groups are either hydrophobic and preferentially interact with the acyl cores of the lipids, or hydrophilic and primarily interact with the buffer. For example, in SMA (Fig. 1b) the maleic acid groups interact with the buffer and the styrene groups interact with the lipids. The important difference is that detergents generally work via replacing lipid association with detergent association (Ilgü et al. 2014), whereas polymers retain a lipid annulus surrounding the protein (Xue et al. 2018). The result is a lipid cluster wrapped by a polymer ‘belt’ that shields the hydrophobic acyl core from interactions with water molecules. These particles typically have a diameter of ~ 10 nm, though the exact diameter is dependent on the polymer used and can increase if proteins are captured during formation (Orwick et al. 2012; Oluwole et al. 2017a; Yasuhara et al. 2017; Bada Juarez et al. 2019).

The ratio of hydrophobic to hydrophilic groups in the SMA polymers can vary and are often combined via an uncontrolled, stochastic polymerisation process. For membrane protein purification, ratios of 2:1 or 3:1 styrene:maleic acid have been most commonly used to form nanoparticles (Scheidelaar et al. 2016; Gupta et al. 2017), and a summary of use cases in the literature can be found within Supplementary Table 3. It is worth noting that there are multiple vendors that provide SMA polymers, which leads to differences in terminology. PolyScience provides polymers with an average molecular weight of either 10 kDa (SMALP 25010P), or 6.5 kDa (SMALP 30010P). Both form nanoparticles that are generally referred to as SMALPs. Meanwhile, the SMA variants that are available from Malvern Cosmeceutics (distributed by Sigma-Aldrich) have an average molecular weight of 9.5 kDa and form nanoparticles that are referred to as Lipodisqs^™^. Whilst this is a distinction in the literature, chemically the polymers can be considered equivalent for their nanoparticle characteristics (Zhang et al. 2015; Hall et al. 2018). The SMA nanoparticles examined in this study are Lipodisqs^™^.

Lipid-polymer nanoparticles have been used in a wide variety of biophysical and structural biology methods. Their smaller correlation times compared to the commonly used model membrane system, large unilamellar vesicles (LUVs), allow for more sensitive measurements in both electron paramagnetic resonance (EPR) and nuclear magnetic resonance spectroscopy (NMR) (Orwick et al. 2012; Orwick-Rydmark et al. 2012; Cuevas Arenas et al. 2016; Sahu et al. 2017). In addition, the retention of native lipids within the nanoparticles has been recently studied using mass spectrometry (Marty et al. 2016; Schmidt et al. 2019; Hoi et al. 2021). These native lipids have even been observed in lipid-polymer nanoparticles through both X-ray crystallography and cryo-electron microscopy (Broecker et al. 2017; Parmar et al. 2018). The nanoparticles also have drug applications, where delivery of the chemotherapy drug doxorubicin has been probed both in vitro and in vivo (Torgersen et al. 2020).

Despite the successes achieved using SMA, the application range of SMALPs or Lipodisqs^™^ is somewhat limited by the chemical properties of the polymer. First, the aromatic styrene groups (Fig. 1b) absorb in the UV range with a peak maximum at 245 nm, which renders them unsuitable for some spectroscopic methods (Rodebush and Feldman 1946). Second, the acid–base behaviour of the carboxylate groups of the maleic acid residues limits the pH range in which nanoparticles can be formed. In addition, the carboxylic acid groups readily bond to divalent cations, which creates sensitivity to even low Mg^2+^ and Ca^2+^ concentrations in buffers (Kopf et al. 2020). These ions are often used for activity assays of membrane proteins, as vital co-factors for proteins such as ATPases (Ryan 1991), or calcium-dependent transporters and ion channels (Van Hook et al. 2019), which limits the use of the SMA-based polymers.

There have been several improvements to the polymer structure that have expanded the chemical properties of the original SMA. An SMA variant featuring a quaternary amine group, SMA-QA, was developed to reduce the polymer sensitivity to divalent cations. This modification also expands the operable pH range, from 6.5–10 to 2.5–10 (Ravula et al. 2018). The styrene moiety has also been replaced to form the diisobutylene and maleic anhydride (DIBMA) polymer (Fig. 1c) (Oluwole et al. 2017b). DIBMA nanoparticles are more tolerant of divalent cations, relative to SMA, and can be used with solubilisation buffers containing up to ~ 10 mM Mg^2+^ or ~ 7.5 mM Ca^2+^ (Oluwole et al. 2017a; Danielczak et al. 2019). However, DIBMA is still limited in regard to pH and cations as it retains the acidic moieties (Fig. 1c). A glycosylated version of DIBMA, Glyco-DIBMA, has recently been developed and shows some promise to reduce the issue of polymer charge upon encapsulated proteins and lipid bilayers (Danielczak et al. 2022). A third type of polymer using polymethacrylate (PMA) (Fig. 1d) has been developed to form lipid-polymer nanodiscs. PMA replaces the acidic groups with a tertiary amine ester and the styrene group with an ester-linked butyl group. PMA is reported to have a broader pH and cation tolerance relative to SMA, its derivatives, and DIBMA (Yasuhara et al. 2017). Similar to SMA and DIBMA, PMA has been shown to effectively solubilise active membrane proteins (Lavington and Watts 2021).

The ideal polymer for forming lipid-polymer nanodiscs depends primarily on the membrane composition, and buffer conditions required for protein solubilisation and function. There are several studies that have characterised various aspects of lipid-polymer nanoparticles, which are mainly focused on SMA variants (Grethen et al. 2017; Hall et al. 2018; Dutta et al. 2019; Gulamhussein et al. 2019). As new polymers have been developed, these have often only been characterized relative to the established SMA polymers. However, there has been no systematic comparison of the commonly used polymers, their efficacy over a broad range of buffer conditions, and the characterisation of nanoparticles formed under consistent conditions. As there are increasing numbers of SMA-variant and novel polymers, a standardised comparative assay is required to easily compare polymer solubilisation efficiency when applied to various buffer and membrane compositions. Here we present a biophysical characterisation of lipid-polymer nanoparticles formed using four commercially available polymers: SMA 3:1 (styrene:maleic acid), SMA 2:1, DIBMA, and PMA. We determine optimal solubilisation conditions qualitatively using lipid-only nanoparticles across a range of pH, salinity and in differing divalent cation concentrations. This characterisation was performed on two membrane models, DMPC and POPC:POPG (3:1). Transmission electron microscopy (TEM) was used to compare the particle size and shape of the different DMPC-polymer nanoparticles.

## Materials and methods

### Polymers

SMA 3:1 and SMA 2:1, with an average molecular weight of 9.5 kDa, which form nanoparticles that are referred to as Lipodisq^™^, were obtained from Malvern Cosmeceutics. DIBMA and PMA were kindly donated by Prof. Sandro Keller and Avanti, respectively. SMA polymers were hydrolysed from the anhydrous form, following a previously published procedure (Gulati et al. 2014). All polymers were resuspended and stored in 20 mM HEPES, 100 mM NaCl, pH 7.4 at a concentration of 100 mg/mL (10% *w/v*).

### Lipid preparation

All lipids used were purchased from Avanti (Birmingham, AL) and stored at – 20 °C at a concentration of 20 mg/mL. DMPC lipid was stored in chloroform, whilst POPC:POPG (3:1 molar ratio) was stored in a 3:1 (*v/v*) chloroform:methanol mixture. Stored lipid was removed as required, and dried under a nitrogen stream, before further desiccation under vacuum to form a dried lipid film. These dried lipid films were then stored at – 20 °C for no more than 12 h prior to use. For samples containing large unilamellar vesicles (LUVs), the lipid film was resuspended in 20 mM HEPES, 100 mM NaCl, pH 7.4 to the desired lipid concentration and incubated at 30 °C for 30 min with gentle agitation. The resuspended lipid was subjected to five freeze–thaw cycles and then extruded at least 30× through 400 nm polycarbonate filters, to form LUVs.

### Spectroscopic methods

Optical density (OD) measurements were recorded within a 96-well plate, using a CLARIOstar plate reader at a wavelength of 350 nm. For each sample, 20 μL of lipid dispersion in distilled H_2_O was aliquoted and diluted with 80 μL of the required buffer to a final lipid concentration of 1 mg/mL (buffers are described in SI Table 1). The samples were agitated at 30 °C for 20 min. Polymer was then added to the desired wells at a 1.5 × (*w/w*) excess and incubated at 30 °C with gentle agitation for 1 h before measurement.

Dynamic light scattering (DLS) was recorded immediately after the measurement of the OD_350_. For each condition, 100 μL of OD_350_ sample was extracted and filtered using a Durapore PVDF 0.22 μm filter. 20 μL of filtrate was measured using a Malvern Zetasizer Nano S. Between 10 and 20 scans at 20 °C were recorded per sample. Hydrodynamic radii were calculated using OmniSize 3.0 software, utilising the ‘mass’ mode.

### Negative stain transmission electron microscopy

Transmission electron microscopy (TEM) was performed on DMPC-polymer nanoparticles formed in 20 mM HEPES, 100 mM NaCl at pH 7.4. 13 μmol of DMPC was removed from a 25 mg/mL chloroform stock solution and subsequently dried under nitrogen. The resulting lipid film was then stored under a high vacuum for at least 4 h, resuspended in 20 mM HEPES, 100 mM NaCl, pH 7.4 to a total volume of 2 mL, and gently agitated at 30 °C for 20 min. 500 μL DMPC fractions per polymer type were suspended with polymer at 1.5 × (*w/w*) and gently agitated for another 20 min at 30 °C. Samples then underwent size exclusion chromatography, using a Superdex 200 increase to remove excess polymer. Purified nanoparticles were concentrated using an Amicon 100 k MWCO spin concentrator to a final calculated lipid concentration of 0.01 mg/mL.

**Fig. 2:**
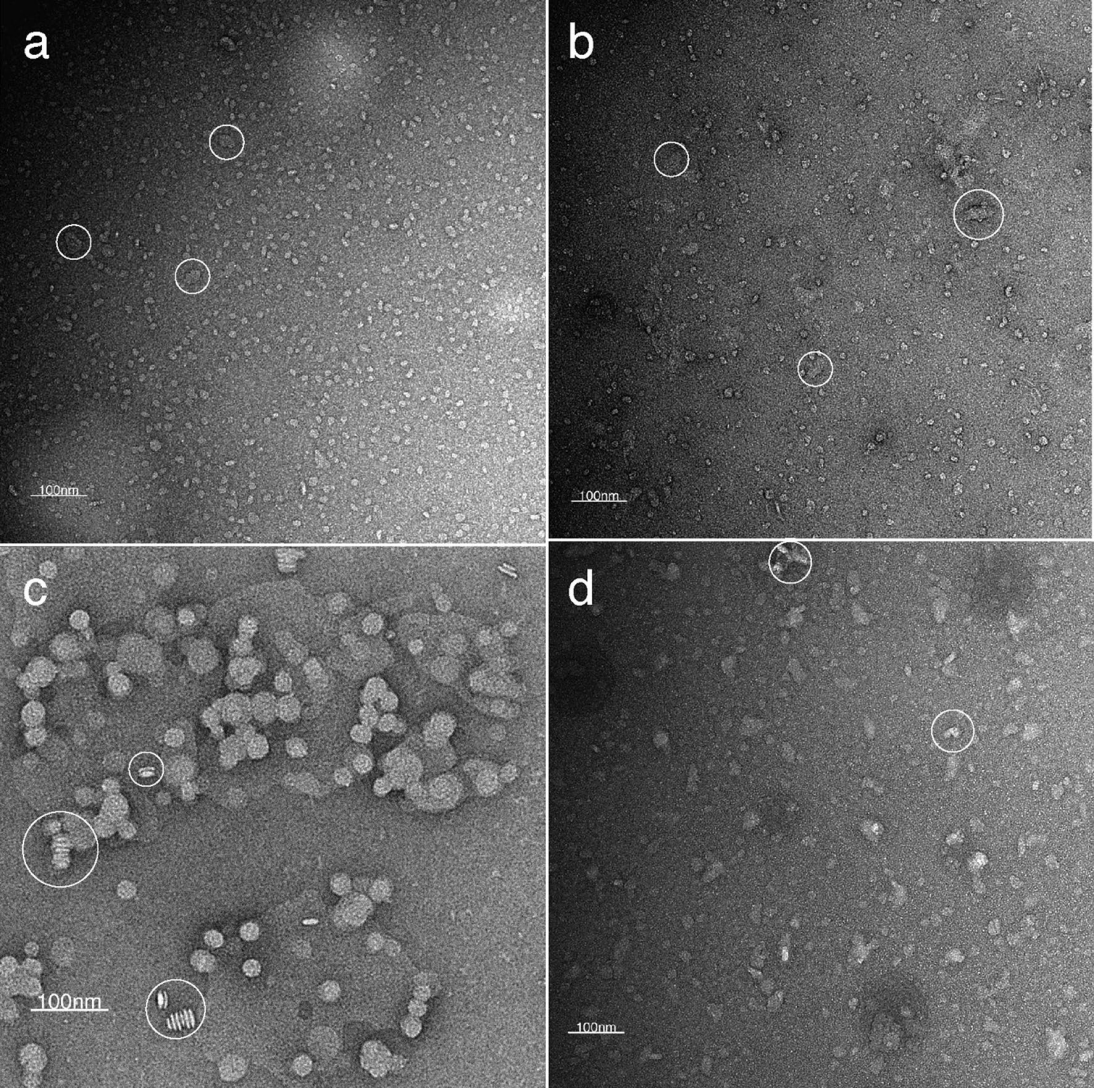
Negative stain transmission electron microscopy images of DMPC-polymer nanoparticles: SMA 3:1 (**a**), SMA 2:1 (**b**), DIBMA (**c**), and PMA (**d**). Nanoparticles were formed by the addition of 1.5% (w/w) polymer to suspended DMPC membranes under optimal conditions (20 mM HEPES, 100 mM NaCl, pH 7.4), followed by size exclusion chromatography to remove excess polymer. White circles highlight abnormal clustering or rouleaux stacking

For TEM of SMA 3:1, SMA 2:1, and PMA nanoparticles (Fig. 2a, b, and d), 3 μL of the sample were incubated on carbon-coated 400-mesh copper grids (glow discharged at 15 mA for 60 s) for 30 s, washed four times with 3 μL of distilled water and stained twice for 15 s with 3 μL of 2% uranyl acetate. Blotting after each incubation step was done at a 45° angle with Whatman filter paper. Images were recorded at ~ 1.5 µm defocus on an FEI Tecnai 12 LaB6 electron microscope (Thermo Fisher Scientific) operated at 120 kV with a Gatan OneView CMOS camera (×30,000 or ×49,000 magnification).

For TEM of DIBMA nanoparticles (Fig. 2c), 3 μL of the sample was absorbed to the clean side of a carbon film on mica and incubated for 30 s, washed several times with 3 μL of distilled water, and stained twice with 2% sodium silico tungstate (SST). Images were taken under low dose conditions (< 10 e-/Å2) with defocus values between 1.2 and 2.5 μm on an FEI Tecnai 12 LaB6 electron microscope at 100 kV accelerating voltage using a Gatan Orius 1000 CCD Camera.

### Electron paramagnetic resonance

DMPC dispersions were prepared with 1-palmitoyl-2-stearoyl-(5-doxyl)-*sn*-glycero-3-phosphocholine (5-PCSL) at a molar ratio of 1:100, and subsequently dried under nitrogen. The resulting lipid film was then stored under a high vacuum for at least 4 h, and resuspended in 20 mM HEPES, 100 mM NaCl at pH 7.4. The samples underwent gentle agitation at 30 °C for 20 min. To form the LUV sample, an aliquot of dispersed lipid was removed and diluted to 100 μL. This was then subjected to five freeze–thaw cycles and extruded at least 30× through 400 nm polycarbonate filters, to form LUVs. The sample was then centrifuged at 30,000g, 4 °C for 30 min, and the pelleted LUVs resuspended to a concentration of 25 mg/mL before insertion into capillary tubes. DMPC dispersions used to form DMPC-polymer nanoparticles were separately aliquoted, and the desired polymer was added at 1.5 × (*w/w*). The DMPC-polymer suspension was then agitated at 30 °C for > 1 h to ensure nanoparticle formation. To remove any unsolubilised membrane, samples were centrifuged at 30,000g, 4 °C for 30 min, with the resulting supernatant (at a final lipid concentration of 25 mg/mL) being inserted into capillary tubes for measurement. Continuous wave electron paramagnetic resonance (cw-EPR) measurements were carried out on a Bruker BioSpin GmbH EMXmicro X-band CW, fitted with a nitrogen cryostat. Prior to measurement, samples were incubated at the desired temperature for 3 min, then recorded for 32 scans at a sweep width of 150 Gauss (*G*) per measurement point. The spin order parameters (*S*) were calculated from the EPR spectra using the following:

$$S= \frac{{A}_{\parallel }-{A}_{\perp }}{{A}_{zz}-(\frac{1}{2})({A}_{xx}+{A}_{yy})}\times \frac{{a\mathrm{^{\prime}}}_{0}}{{a}_{0}}$$

where

$${a\mathrm{^{\prime}}}_{0}= \left( {\frac{1}{3}} \right)(A_{{xx}} + A_{{yy}} + A_{{zz}} )$$

and

$${a}_{0}=\left( {\frac{1}{3}} \right)({A}_{\parallel }+2{A}_{\perp })$$

where *A*_||_ is the hyperfine splitting parallel to the membrane normal, *A*_⊥_ is the hyperfine splitting perpendicular to the membrane normal*. A*_*xx*_, *A*_*yy*_, and *A*_*zz*_ are the principal hyperfine splitting corresponding to the molecular axis of the spin label at 5.9, 5.4, and 32.9 Gauss, respectively (Marsh and Watts 1981).

## Results and discussion

### Qualitative study of membrane solubilisation by four polymers

**Fig. 3:**
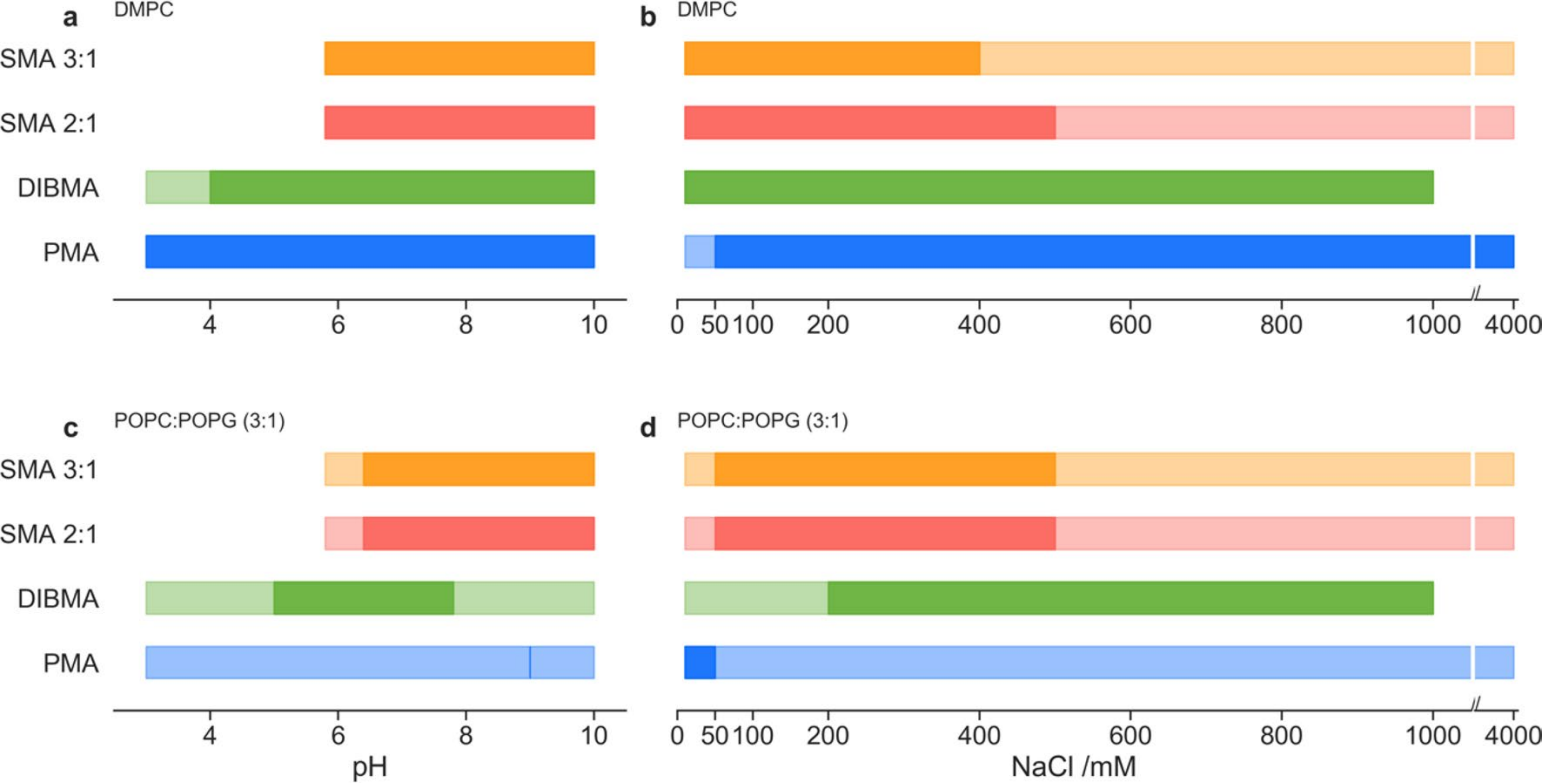
Qualitative range of membrane solubilisation for: DMPC membranes; across a pH range at a constant salt concentration of 100 mM (**a**); across a NaCl concentration gradient/range (**b**), and POPC:POPG (3:1); across a pH range (**c**); across a NaCl concentration gradient/range (**d**). Ranges are derived from DLS measurements (lighter colour) where nanoparticles were detected (where 2 nm < R_H_ < 10 nm). Darker colour range is indicative of OD_350_ < 2 after polymer addition, indicating significant membrane solubilisation

Two membrane models, DMPC and POPC:POPG (3:1), were used to study the impact of both the polymer and the buffer conditions on solubilisation. DMPC has often been chosen as the membrane of choice for nanoparticle studies, while POPC:POPG (3:1) was used as a more complex model for bacterial membranes. Lipid suspensions were prepared in different buffers that cover a range of either pH, salt concentration, or divalent cation concentrations. Each buffer condition was tested with the four polymers, using both Dynamic Light Scattering (DLS) and Optical Density at 350 nm (OD_350_). The DLS measurement determined in which conditions the addition of polymer led to nanoparticle formation, through the identification of particles with a radius (*R*_H_) between 2 and 10 nm. A decrease in the OD_350_ (due to a reduction in particle size and therefore elastic light scattering) indicated the removal of particles with a diameter over 175 nm, which in turn implies membrane solublization into lipid-polymer nanoparticles. Hence, OD_350_ measurements were used to detect when a significant proportion of the lipid suspension had been solubilised by the added polymer. Together, these orthogonal measurements served to identify conditions that led to lipid solubilisation and nanoparticle formation (Fig. 3).

Generally, DLS was able to determine the presence of nanoparticles in a broader range of conditions, relative to OD_350_. However, DLS is not a quantitative measurement of the number of nanoparticles formed. Hence, using the combined measurements, we interpreted conditions with a positive DLS measurement, but no reduction in OD_350_, as non-optimal solubilisation conditions, whereas conditions with both, a positive detection by DLS and a reduction in OD_350_, were labelled as optimal solubilisation conditions.

For the DMPC lipids, samples covering a pH range from 3 to 10 were tested (Fig. 3a). All polymers were found to optimally solubilise lipids at a pH > 6.4. SMA was found to be poorly solubilizing below this pH. The lower pH limit was expected, as it has been reported that SMA precipitates in buffers below pH 6 (Scheidelaar et al. 2016; Kopf et al. 2020). In contrast, both DIBMA and PMA had broader solubilisation pH ranges that reached as low as 4 and 3, respectively. Furthermore, tolerance to salinity was examined with salt concentrations from 0 mM to 4 M (Fig. 3b). While DLS detected nanoparticles over all salt concentrations with the SMA polymer, the OD_350_ measurements indicated that the optimal salt concentration was below 400 mM and 500 mM for SMA 3:1 and SMA 2:1, respectively. DIBMA showed optimal solubilisation up to 1 M salt concentration. The PMA polymer showed the largest range, achieving solubilisation from 50 mM up to the highest salt concentration tested, 4 M. The low tolerance for < 50 mM NaCl is most likely explained by a requirement for some counter ions for PMA solvation in the aqueous buffer, without which PMA aggregates or becomes inactive. This can be more clearly observed in SI Fig. 2a, where the OD_350_ is comparable to the pre-solubilised membrane result. The high salt tolerance observed for PMA may be explained by the lower hydrophobicity of PMA, as compared to DIBMA and SMA (Sadeghi and Jahani 2012).

More narrow optimal solubilisation ranges of pH and salt concentration were observed for the more complex POPC:POPG (3:1) membrane model. This is most likely due to the increased charge repulsion between membrane and the solubilising polymer, as well as the difference in lateral pressure between the fully-saturated DMPC and the singly-unsaturated POPC:POPG membrane. Such an interaction would explain the overall decrease in optimal solubilisation conditions (measured by OD_350_), but not the decrease in nanoparticle formation (as measured by DLS). Both SMA polymers were found to solubilise lipids only above a pH of 5.8 (Fig. 3c). A broader pH range was observed for DIBMA, which showed optimal solubilisation above a pH of 4 (OD_350_), while DLS showed that nanoparticles were present over the full pH range. PMA also showed nanoparticle formation over the entire range by DLS, but optimal solubilisation was only observed with pH 9. The lack of solubilisation with PMA is likely due to its smaller hydrophobic group (compared to SMA and DIBMA) which would affect polymer-membrane interactions for the charged membrane model. Additionally, the salt concentration for the pH range was set to 100 mM, but the later performed salinity screen for PMA with the POPC:POPG (3:1) model membrane showed optimal solubilisation only at NaCl concentrations up to 50 mM (Fig. 3d). This apparent optimal window is most likely an artefact of the graphical representation, as in the raw OD_350_ data (SI Fig. 2b), the change in OD compared to the pre-solubilised membrane is minimal. It is unlikely, that the change in optimal salinity for PMA between DMPC and POPC:POPG is due to the anionic charge of the POPC:POPG membranes, as a similar trend is observed also for uncharged POPC:POPE (3:1) membranes (SI Fig. 5). For both SMA variants it is shown that nanoparticles can form over the entire range of salt concentrations, but optimal solubilisation is only achieved between 50 and 500 mM. The optimal conditions for DIBMA are increased to a minimum of 200 mM, up to 1 M. The requirement for salinity when solubilising charged membranes has been described previously by both Tanaka et al*.* (2020) and Kopf et al*.* (2020) for SMA polymers, and similar patterns can be observed here for both DIBMA and PMA, although only for the DMPC membrane in the case of PMA. The lower salinity required by SMA for significant solubilisation compared to DIBMA and PMA may be due to the increased hydrophobicity of the styrene groups compared to the other polymers.

The ranges described here for pH and salinity are consistent with the conditions described in the literature for protein solubilisation by the polymers examined in this work. A pH between 7.4 and 8 is most common for detergent-free protein solubilisation (SI Table 3), and this is well within the optimal range as shown by Fig. 1a and c. Similarly, a higher salinity for charged membranes (as observed in Fig. 1d) mirrors the common salinity of 200–400 mM in literature protocols (SI Table 3). The ranges shown in this work indicate that the optimal conditions for protein solubilisation can be extended beyond those commonly observed in the literature. However, as shown by Kopf et al., solubilisation of membrane proteins by polymers is partially dependent on the membrane protein in question (Kopf et al. 2020), meaning the values observed for the lipid-only solubilization may vary slightly for membrane protein purification.

**Fig. 4:**
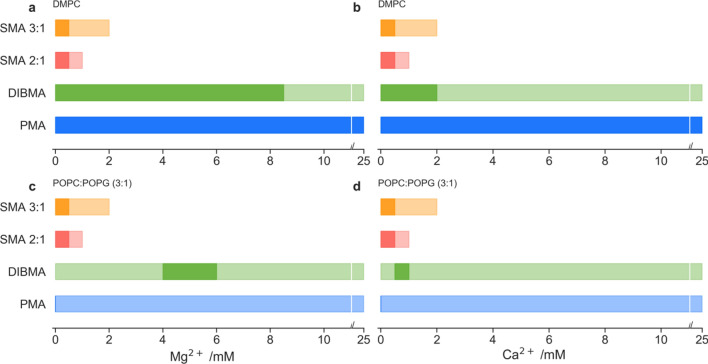
Qualitative range of membrane solubilisation for: DMPC membranes; across a magnesium concentration from 0 to 25 mM with a constant ionic concentration of 100 mM (**a**); across a calcium concentration from 0 to 25 mM with a constant ionic concentration of 100 mM (**b**), and POPC:POPG (3:1); across a magnesium concentration from 0 to 25 mM with a constant ionic concentration of 100 mM (**c**); across a calcium concentration from 0 to 25 mM with a constant ionic concentration of 100 mM (**d**). Ranges are derived from DLS measurements (lighter colour) where nanoparticles were detected (where 2 nm < R_H_ < 10 nm). Darker colour range is indicative of OD_350_ < 2 after polymer addition, indicating significant membrane solubilisation

The same experimental setup was used to probe the solubilisation potential in the presence of either Ca^2+^ or Mg^2+^ divalent cations (Fig. 4). Here the assay was adapted to ensure the total ionic strength at each measurement was kept constant. Generally, SMA was intolerant of divalent cations with identical limits for the solubilisation of both the DMPC and POPC:POPG (3:1) membrane models. For both Mg^2+^ and Ca^2+^, nanoparticles were detected in solution by DLS, up to divalent concentrations of 2.0 mM and 1.0 mM for SMA 3:1 and 2:1, respectively. However, the OD_350_ measurements showed that successful solubilisation was only achieved up to divalent concentrations of 0.5 mM. These findings mirror the observations of Kopf et al*.* (2020) and confirm the visual precipitation of the SMA polymer observed above this concentration, rather than a non-optimal solubilisation.

In contrast, both DIBMA and PMA were seen to form nanoparticles under greater divalent cation concentrations. In both the DMPC and POPC:POPG (3:1) membrane models, DLS measurements detected nanoparticles over the entire concentration range. However, the OD_350_ measurements again showed a narrowing of optimal range that allowed for successful solubilisation of the charged membrane model compared to the DMPC membranes (Fig. 4a and b vs c and d). In the DMPC model DIBMA was able to achieve optimal solubilisation up to a maximum divalent concentration of 8.5 mM and 2.5 mM for Mg^2+^ and Ca^2+^ ions, respectively. The increased tolerance by DIBMA to divalent cations over SMA is possibly due to the higher maleic acid content per polymer length (DIBMA is an alternating polymer of diisobutylene and maleic acid whilst SMA 3:1 and 2:1 are random copolymers of 3:1 and 2:1 styrene:maleic acid, respectively). In the more complex POPC:POPG (3:1) model, DIBMA showed regions of optimal solubilisation between 4.0 and 6.0 mM and 0.25–0.50 mM for Mg^2+^ and Ca^2+^ ions, respectively (Fig. 4c and d). This has been previously reported by Danielczak et al. (2019) and hypothesized to be due to the partial association of divalent cations to excessive maleic acid groups along DIBMA’s length, increasing polymer-membrane association. PMA showed optimal solubilisation over all conditions for the DMPC membrane, while it was unable to achieve optimal solubilisation for the POPC:POPG (3:1) membrane model. This could be due to an overall positive charge at the membrane surface caused by the association of divalent cations with the anionic lipid POPG, thus reducing the attraction of the positively charged PMA. As shown in Fig. 1, PMA is a relatively weak solubilising polymer, so any increase in charge repulsion between PMA and the membrane would drastically reduce the solubilisation efficiency measured by OD_350_.

The work in this section shows that lipid-polymer nanoparticles can form over a broad range of conditions, however, achieving optimal, i.e. efficient, solubilisation of lipids was only possible over a smaller range of conditions. It is worth noting that the temperature used during the extraction is another important factor that we have not investigated in this study. This has recently been highlighted by Kopf et al*.,* who indicated that a fast, warm extraction can increase the efficiency of polymer solubilisation without significantly affecting membrane fluidity of the formed nanoparticles. This implies that a warmer extraction does not necessarily have deleterious effects on the membrane protein of interest (Kopf et al. 2020). Furthermore, whilst our work has been comprehensive for assessing buffer conditions for the solubilisation of model membranes, the additional complexity of native membranes combined with downstream protein purification techniques also affect the choice of polymer for protein solubilisation. For example, the presence of SMA and DIBMA has been shown to affect purification steps, in particular Ni–NTA binding (Scheidelaar et al. 2016; Qiu et al. 2018; Lavington and Watts 2021).

Despite the narrow applicability of SMA that we have just demonstrated, SMA remains the most popular polymer when screening the literature. Its popularity is most likely due to the high yields of solubilisation that can be achieved at optimal buffer conditions, due to the styrene’s high hydrophobicity that drives membrane-polymer interactions (Bjørnestad et al. 2021). Additionally, optimal solubilisation conditions for SMA are often similar to well-established detergent-based solubilisation conditions, allowing for relatively easy optimisation of conditions when switching to polymer-based solubilisation. Meanwhile, the relatively low use of DIBMA-based solubilisations is most likely due to two factors; (i) the lower hydrophobicity of the isobutyl groups leads to a lower yield of protein-containing DIBMA nanoparticles during membrane protein extraction, and (ii), DIBMA has a smaller optimal solubilisation window for charged membranes than SMA. Together, these factors lead to a preference for the stronger solubilising SMA over DIBMA in similar solubilisation conditions, with the exception of cases where divalent cations are required for protein activity. PMA, which we demonstrate has high stability in the buffer conditions examined in this work, presumably is the second choice for classical, physiological solubilisation conditions. However, it is likely to find popularity in its ability to form lipid-PMA nanoparticles in either acidic or extreme high salt solubilisation conditions, such as in the solubilisation of membrane proteins from extremophiles. This is due to polymers, such as SMA and DIBMA being observed to precipitate or become non-functional under those conditions as demonstrated in this work, whereas no such precipitation was observed with PMA samples.

Finally, the prevalence of SMA over other polymers is also likely due to being related to the relative availability of SMA. As it was the first described, and for a long time only commercially available polymer, it was the only viable option for polymer-based solubilization attempts. However, with a fast-growing number of both general competitors such as DIBMA, and specialised SMA-variants becoming available, it is likely that this advantageous position in the literature will change in the coming years.

### Nanoparticle structure analysis through transmission electron microscopy (TEM)

To ensure that the identified solubilisation conditions yield characteristic homogenous nanodiscs, samples derived from the optimal conditions for the DMPC membrane model (20 mM HEPES, 100 mM NaCl and a pH of 7.4) were visualised using negative stain transmission electron microscopy (TEM). The images showed most nanoparticles to be distinct, coin-shaped particles in the range of 12–25 nm in diameter (SI Fig. 13, SI Table 2). Some clustering and stacking, known as ‘rouleaux’ stacks, were also observed. These stacks have been previously described as artefacts caused by the phosphatidylcholine headgroups and charged inorganic crystals from sample staining (Dominguez Pardo et al. 2017).

The average diameters of each polymer nanoparticle were extracted from the micrographs, taking the major axis for each measurement each time. The average diameters were 14.1 ± 2.6 nm for SMA 3:1, 13.7 ± 2.4 nm for SMA 2:1, 24.5 ± 3.8 nm for DIBMA and 18.8 ± 4.2 nm for PMA (SI Table 2). The observed diameters for SMA are within the measurement error of the DLS analysis performed in the previous Section (8.2 ± 4.4 nm for SMA 3:1, 8.6 ± 3.7 nm for SMA 2:1). However, a variation in the average diameter determined by TEM and DLS was seen for DIBMA (24.5 ± 3.8 nm vs 11.9 ± 4.9 nm) and PMA (18.8 ± 4.2 nm vs 9.3 ± 3.4 nm). The TEM diameter for PMA is in line with the TEM diameter measured by Yashuhara et al. (17 nm), however, they have not disclosed the equivalent DLS diameter in their publication (Yasuhara et al. 2017). The overall discrepancy between TEM and DLS diameters might be due to the staining protocol used in our study which has been previously optimised for SMA nanoparticles (Orwick et al. 2012) and potentially affects the recorded size of DIBMA and PMA nanoparticles.

### Lipid dynamics of DMPC-polymer nanoparticles by cw-EPR

**Fig. 5:**
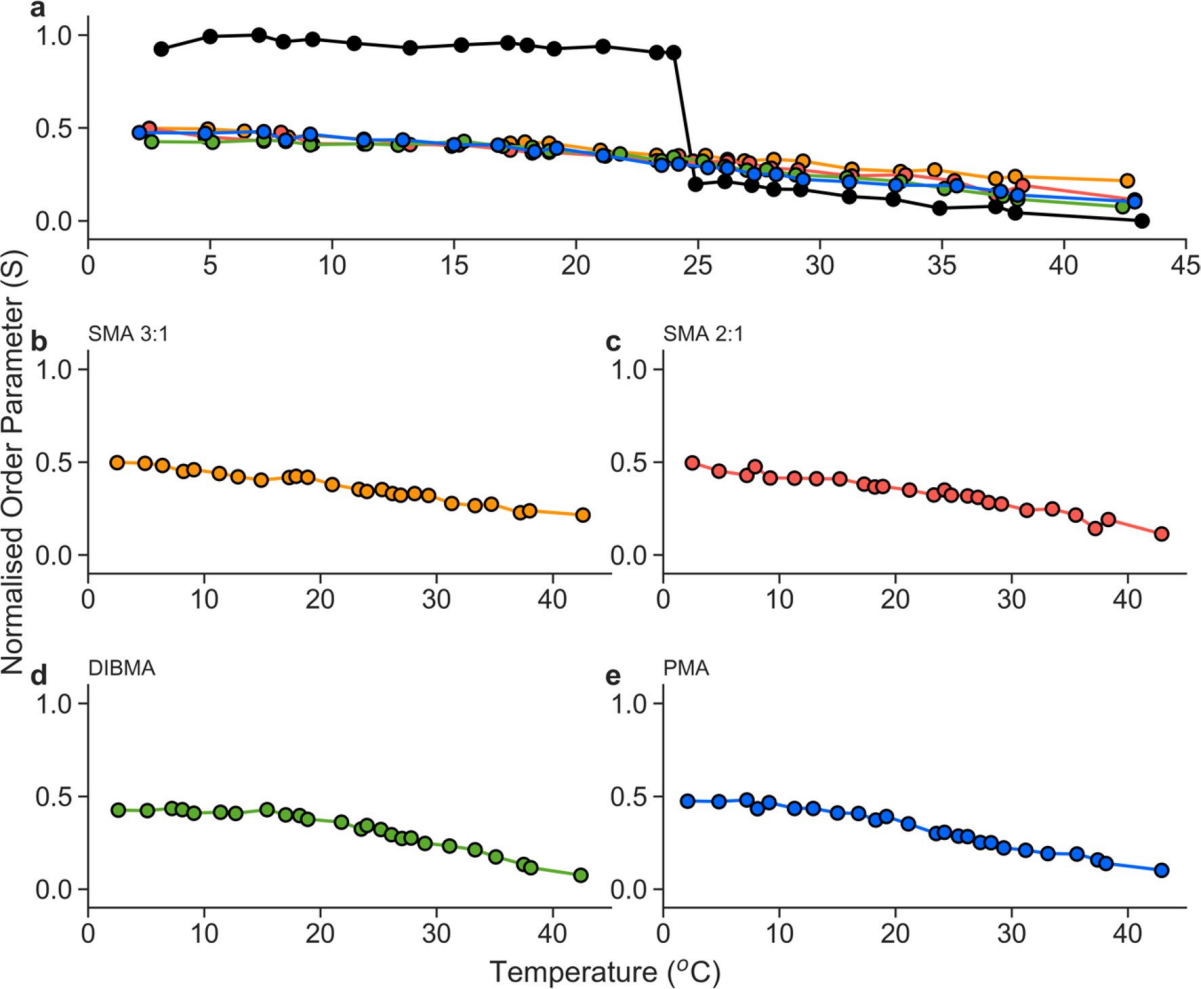
Normalised order parameter (*S*) of lipid membrane generated from cw-EPR measurement of 1% (molar) 5-PCSL in DMPC-polymer nanoparticles formed by the addition of 1.5% (w/w) polymer to suspended DMPC membranes under optimal conditions (20 mM HEPES, 100 mM NaCl, pH 7.4), followed by size exclusion chromatography to remove excess polymer. DMPC LUVs were generated in the absence of polymer, using freeze–thaw and extrusion methods. **a** Order parameter of 400 nm DMPC LUVs (black), SMA 3:1 (orange), SMA 2:1(red), DIBMA (green), and PMA (blue) normalised to LUV order parameter. Isolated normalised order parameters of SMA 3:1 (**b**), SMA 2:1 (**c**), DIBMA (**d**), and PMA (**e**) nanoparticles

The main phase transition observed in DMPC large unilamellar vesicles (LUVs) at 24 °C is visible in cw-EPR measurements (Fig. 5a), using 5-PCSL labelled lipids. The cw-EPR measurement showed a rapid decrease in lipid packing in the LUV sample, detectable through the decrease in order parameter (*S*), as the lipid converted from the gel phase to the fluid phase. The nanoparticle samples did not experience the same degree of the order shown in the LUVs at low temperatures (in the gel phase). Nor did they exhibit the high level of mobility seen at higher temperatures within the DMPC LUVs (in the fluid phase).

Figure 5b–e show the individual change in order parameter (*S*) of each lipid-polymer nanoparticle, normalised to the order parameter of the LUV control. It is possible to see slight differences in the rate of order parameter reduction with temperature. Figure 5b and c representing SMA 3:1 and 2:1, respectively, show a slow linear reduction in order parameter corresponding to increased membrane fluidity with temperature. Both SMA nanoparticles also generally show increased packing with higher normalised order parameters (*S*) at each temperature relative to PMA and DIBMA nanoparticles. DMPC-DIBMA nanoparticles, shown in Fig. 5d, show a plateau in membrane fluidity until ~ 15 °C. This plateau implies the presence of a slight phase transition around 15 °C before decreasing linearly as seen in the DMPC-SMA nanoparticles. Similarly, Fig. 5e shows an inverse sigmoidal decrease in fluidity between 10 and 30 °C, indicating a subtle phase change in DMPC-PMA nanoparticles.

The 5-PCSL labelled lipids did not show any point of rapid change in fluidity over the entire temperature range in any of the polymer-lipid nanoparticles (Fig. 5b–e). Instead, the nanoparticles showed a broad increase in fluidity with increasing temperature. This agrees with previous EPR measurements of DMPC-SMA 3:1 nanoparticles relative to DMPC LUVs (Orwick et al. 2012). The loss of the sharp phase transition usually observed in LUV systems is indicative of not only the loss of a large cooperative phase unit but also the reduction of acyl chain reordering during the gel-to-fluid phase transition. This linear trend has also been observed in some peptide-based nanodiscs (Anada et al. 2021), and therefore is likely due to a combination of this smaller cooperative unit and the higher lateral pressure within the nanoparticles due to the polymer or peptide belt. For polymer nanoparticles, the increased association of hydrophobic groups from the polymer with the acyl core of the membrane may also affect this fluidity further (Orwick et al. 2012).

The cw-EPR experiments highlight a difference in the phase behaviour of the DMPC bilayer in LUVs and in each of the lipid-polymer nanoparticles. This observation adds to the previous studies of lipid fluidity in SMA- and DIBMA-bound nanoparticles, whilst revealing novel findings for the DMPC-PMA nanoparticles (Orwick et al. 2012; Colbasevici et al. 2020; Hoffmann et al. 2021). These findings are important to consider when determining whether these lipid-polymer nanoparticle systems could be used for membrane protein solubilisation, as membrane fluidity in polymer-based nanodiscs has been implied to affect protein activity (Colbasevici et al. 2020; Voskoboynikova et al. 2021; Szundi et al. 2021).

## Conclusions

The ever-increasing number of polymers that can generate lipid-polymer nanoparticles will inevitably expand the number of membrane proteins able to be solubilised and subsequently characterised. However, the identification of optimal solubilisation conditions is often empirical – particularly for new users of these polymers. Here we have shown that easily accessible biophysical methods can be used to perform a broad characterisation to determine the optimal solubilisation conditions for a given polymer and membrane model prior to large-scale purification attempts. Our study is one of the broadest comparisons of lipid-polymer nanoparticle formation in a single study, with additional comparison from various protocols in the literature (summarised in SI Table 3).

Our investigations demonstrate that buffers mimicking physiological conditions (pH 7.4 and salt concentrations of ~ 200 mM) are the optimal starting point for the solubilisation of both membrane models with all polymers studied. These findings corroborate the common use of these conditions in the literature protocols, but also highlight that optimal solubilisation conditions are achievable beyond the narrow ranges currently described in the literature. Furthermore, if divalent cations are required for protein activity, then either DIBMA or PMA should be used instead of the divalent cation-sensitive SMA polymers. Similarly, when less common solubilisation conditions are required, such as low pH or high salt, our studies identify PMA as the optimal polymer to start with, alongside (beyond the scope of this paper) the modified SMA variants SMA-QA or SMI which have been shown to possess a similar insensitivity to low pH and high salt concentrations (Hall et al. 2018; Ravula et al. 2018). The further development polymers such as SMA-QA (Ravula et al. 2018), Sulfo-SMA/Sulfo-DIBMA (Glueck et al. 2022), and Glyco-DIBMA (Danielczak et al. 2022) highlights the requirement for standardised characterizations, such as those presented in this work.

Finally, this work shows for the first time a characterisation of solubilisation conditions for PMA in direct comparison to other polymers as well as reporting lipid-fluidity in DMPC-PMA nanoparticles. We show that lipids within these lipid-polymer nanoparticles do not experience a native-like cooperative phase transition as in LUV, but instead experience a broadening of the gel-to-fluid phase transition. This has been similarly observed by Hoffman et al. via EPR, as well as by Orwick et al*.* by both EPR and DSC for SMA (Orwick-Rydmark et al. 2012; Hoffmann et al. 2021). However, measurement of DSC in DIBMA nanoparticles shows a more native-like gel-to-fluid transition (Oluwole et al. 2017b), and the results presented in this work may show this enthalpic transition is concurrent with a more fluid bilayer in DIBMA nanoparticles. Nevertheless, measurement by EPR indicate that lipids within these lipid-polymer nanoparticles generally experience a more restricted ‘fluid-like’ state across a broader temperature range due to the smaller cooperative unit, and the increased lateral pressure and (in the case of SMA) interaction of the acyl core with hydrophobic groups of the polymers used. It is therefore likely that proteins within these systems experience a higher degree of lateral pressure than other membrane models, potentially leading to the higher degree of stability observed for proteins in lipid-polymer nanoparticles over other model systems such as micelles and LUVs (Van Den Brink-Van Der Laan et al. 2004).

## Supplementary Information

Below is the link to the electronic supplementary material.

Supplementary file1 (DOCX 5828 KB)

## Data Availability

Data can be requested by contacting the corresponding author.

## Code availability

A portion of the code used for plotting can be obtained at https://github.com/HSawczyc/biophysics2python, additional code may be requested by contacting the corresponding author.
