# Supplementary material for: A comparative characterisation of commercially available lipid-polymer nanoparticles formed from model membranes
Source: Eur Biophys J. 2023 Feb 14;52(1-2):39–51. doi: 10.1007/s00249-023-01632-5 (PMC10039845; doi:10.1007/s00249-023-01632-5)
Supplement: Supplementary file 1 — Supplementary file1 (DOCX 5828 KB) [file 249_2023_1632_MOESM1_ESM.docx]

**Supplementary Information**

for

A comparative characterization of commercially available lipid-polymer nanoparticles

Henry Sawczyc^1*^, Sabine Heit^1^, Anthony Watts^1*^

^1^ Department of Biochemistry, University of Oxford, South Parks Road, OX1 3QU, Oxford, UK.

* Correspondence should be addressed to: henry.sawczyc@gmail.com or anthony.watts@bioch.ox.ac.uk

**Contents**

[Buffers used for membrane solubilisation assays 2](#_Toc68635948)

[OD measurements 4](#_Toc68635949)

[DLS data 9](#_Toc68635950)

[EPR Spectra 10](#_Toc68635951)

[A. DMPC LUV (1% molar 5-PCSL) 11](#_Toc68635952)

[B. DMPC-SMA 3:1 nanoparticles (1% molar 5-PCSL) 12](#_Toc68635953)

[C. DMPC-SMA 2:1 nanoparticles (1% molar 5-PCSL) 13](#_Toc68635954)

[D. DMPC-DIBMA nanoparticles (1% molar 5-PCSL) 14](#_Toc68635955)

[E. DMPC-PMA nanoparticles (1% 5-PCSL) 15](#_Toc68635956)

[TEM nanoparticle measurements 16](#_Toc68635957)

[Summary of published extraction conditions 18](#_Toc68635958)

[Enthalpy values of DSC 20](#_Toc68635977)

[References for the SI 21](#_Toc68635978)

#

# Buffers used for membrane solubilisation assays

All buffers were generated as a 100 mL stock, with a total concentration of 0.125 M. This concentration of buffer components was chosen to achieve a final ionic strength of 0.1 M for the aqueous lipid film (resuspended in dH2O) diluted in the desired buffer. The different buffering salts used for pH titration are shown in Table 1.

NaCl, Mg2+, Ca2+ stocks were buffered with 10 mM HEPES to a pH of 7.4 from a 0.5 M stock solution. The desired ionic concentration was obtained with measured aliquots of 5 M NaCl stock solution. Divalent cation titration buffers were obtained from master stocks of magnesium chloride (1 M MgCl2), and calcium chloride (1 M CaCl2) and had a consistent ionic strength of 0.125 M throughout the divalent cation titration range, with a final ionic strength of 0.1 M during measurement.

**Table 1:** Summary of all pH buffer stock mixtures used for OD350 solubilisation assays. Stocks were prepared at 1.25x concentration, to account for the dilution required for lipid dispersion addition. Ionic strength is reported as final concentration (the concentration found within the plates during measurement of OD350). * denotes the ionic strength for Tris calculated using online calculator tool (https://www.liverpool.ac.uk/pfg/Tools/BuferCalc/Buer.html)

| pH value | Buffer components | Calculated ionic strength (M) |
| --- | --- | --- |
| 3.0 | 0.33 g Sodium citrate dihydrate, 2.19 g Citric acid | 0.9 |
| 4.0 | 1.24 g Sodium citrate dihydrate, 1.59 g Citric acid | 0.9 |
| 5.0 | 2.12 g Sodium citrate dihydrate, 1.02 g Citric acid | 0.9 |
| 6.0 | 0.28 g K2HPO4, 1.50 g KH2PO4 | 0.9 |
| 6.8 | 1.00 g K2HPO4, 0.92 g KH2PO4 | 0.9 |
| 7.0 | 1.17 g K2HPO4, 0.79 g KH2PO4 | 0.9 |
| 7.2 | 1.34 g K2HPO4, 0.65 g KH2PO4 | 0.9 |
| 7.4 | 1.52 g K2HPO4, 0.52 g KH2PO4 | 0.9 |
| 7.6 | 1.69 g K2HPO4, 0.38 g KH2PO4 | 0.9 |
| 7.8 | 1.86 g K2HPO4, 0.25 g KH2PO4 | 0.9 |
| 8.0 | 2.03 g K2HPO4, 0.11 g KH2PO4 | 0.9 |
| 8.5 | 1.514 g Tris-HCl | 0.016* |
| 9.0 | 1.514 g Tris-HCl | 0.005* |
| 10.0 | 0.49 g NaHCO3 , 0.71 g Na2CO3 | 0.4 |

# OD measurements


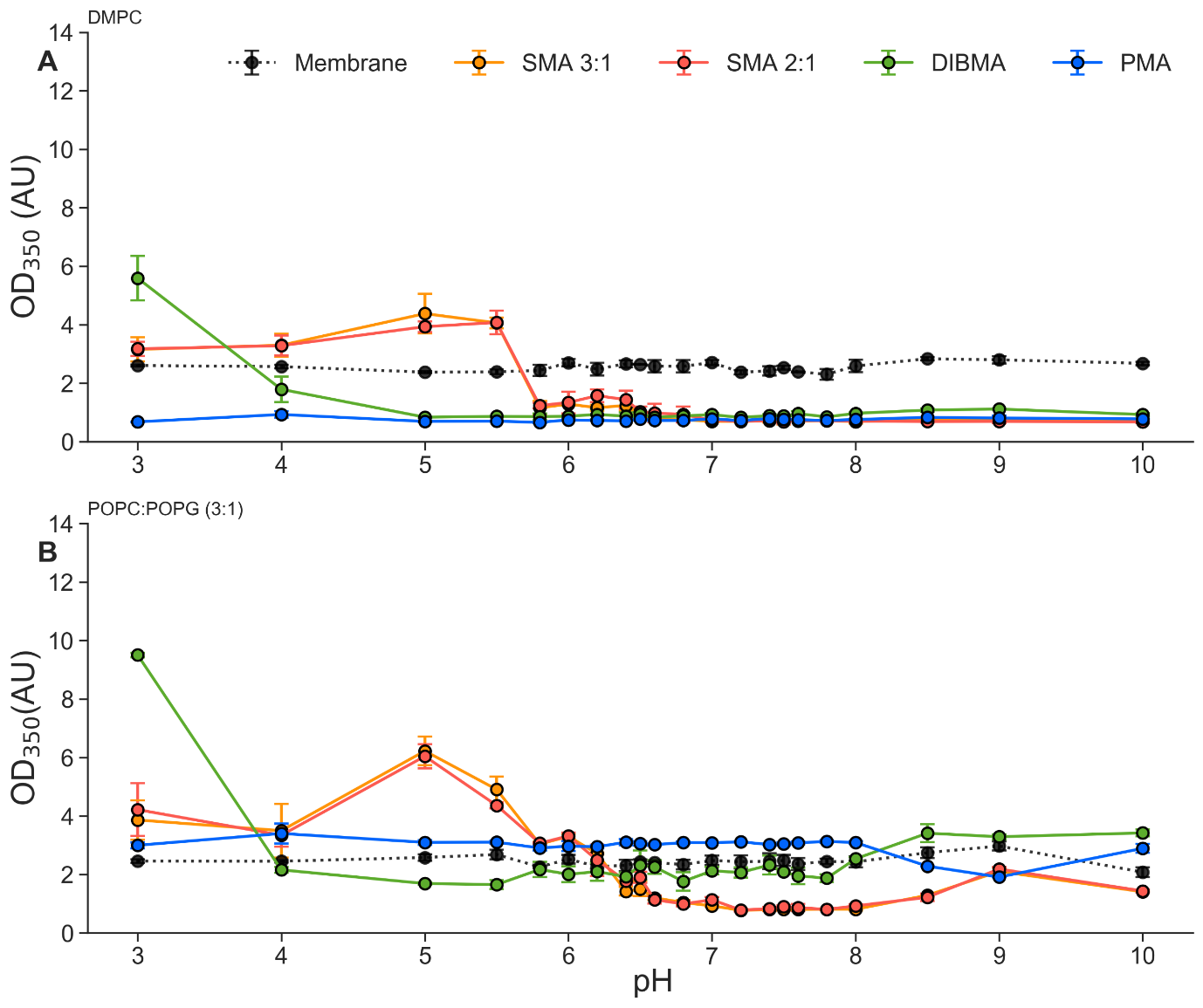


**SI Figure 1** – OD_350_ of pH screening. Absorbance at 350 nm for (A) DMPC membranes and (B) POPC:POPG (3:1) membranes, black line shows absorbance of membrane prior to polymer addition (averaged for all polymer samples). Polymers (SMA 3:1 – orange, SMA 2:1 – red, DIBMA – green, PMA – blue) were added at 1.5*x* membrane weight (*w/w*), to a final concentration of 1.5%, and incubated at 37^o^C for 1 hour with gentle agitation.


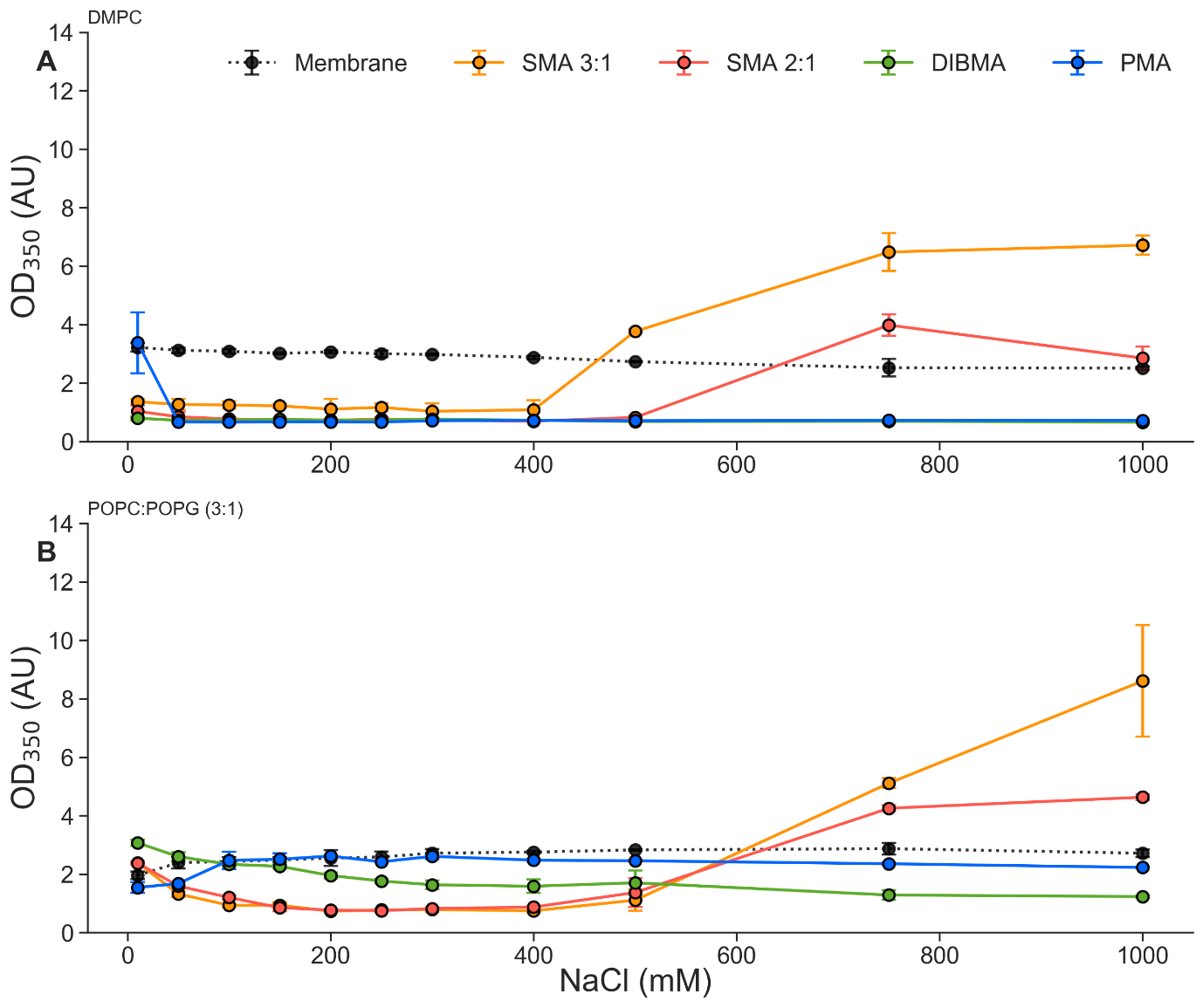


**SI Figure 2** – OD_350_ of NaCl screening. Absorbance at 350 nm for (A) DMPC membranes and (B) POPC:POPG (3:1) membranes, black line shows absorbance of membrane prior to polymer addition (averaged for all polymer samples). Polymers (SMA 3:1 – orange, SMA 2:1 – red, DIBMA – green, PMA – blue) were added at 1.5*x* membrane weight (*w/w*), to a final concentration of 1.5%, and incubated at 37^o^C for 1 hour with gentle agitation.


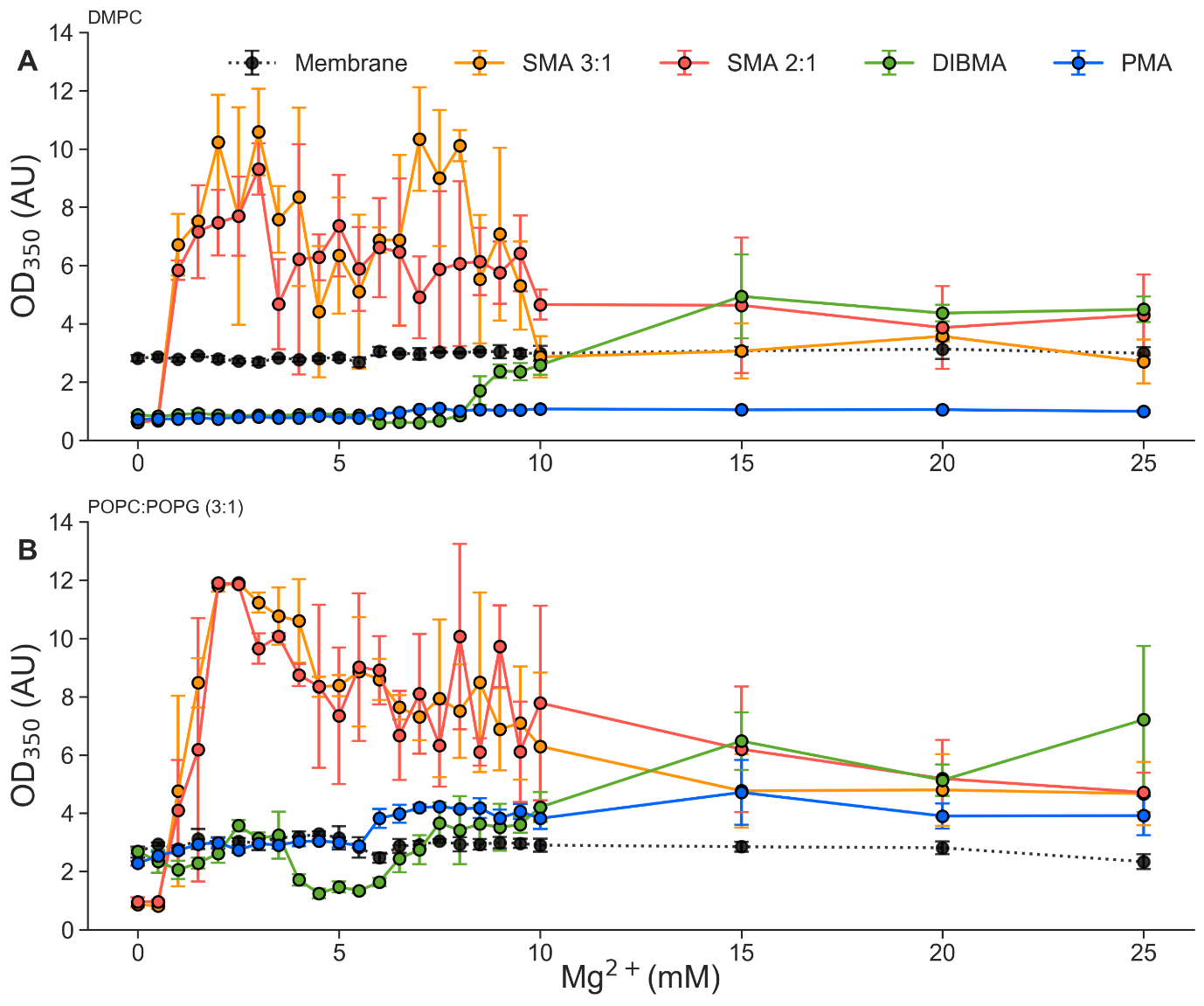


**SI Figure 3** – OD_350_ of Mg^2+^ screening. Absorbance at 350 nm for (A) DMPC membranes and (B) POPC:POPG (3:1) membranes, black line shows absorbance of membrane prior to polymer addition (averaged for all polymer samples). Polymers (SMA 3:1 – orange, SMA 2:1 – red, DIBMA – green, PMA – blue) were added at 1.5*x* membrane weight (*w/w*), to a final concentration of 1.5%, and incubated at 37^o^C for 1 hour with gentle agitation.


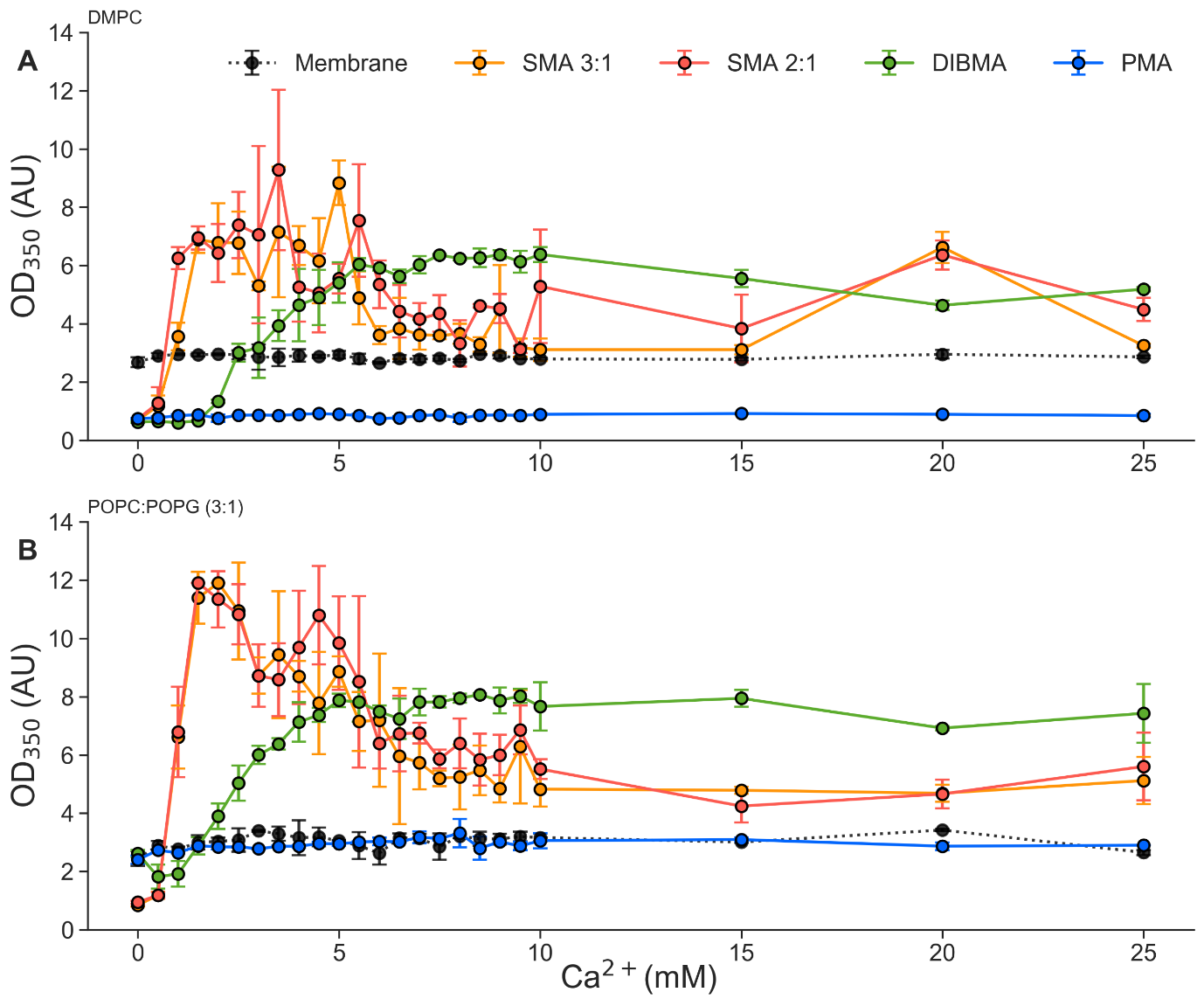


**SI Figure 4** – OD_350_ of Ca^2+^ screening. Absorbance at 350 nm for (A) DMPC membranes and (B) POPC:POPG (3:1) membranes, black line shows absorbance of membrane prior to polymer addition (averaged for all polymer samples). Polymers (SMA 3:1 – orange, SMA 2:1 – red, DIBMA – green, PMA – blue) were added at 1.5*x* membrane weight (*w/w*), to a final concentration of 1.5%, and incubated at 37^o^C for 1 hour with gentle agitation.


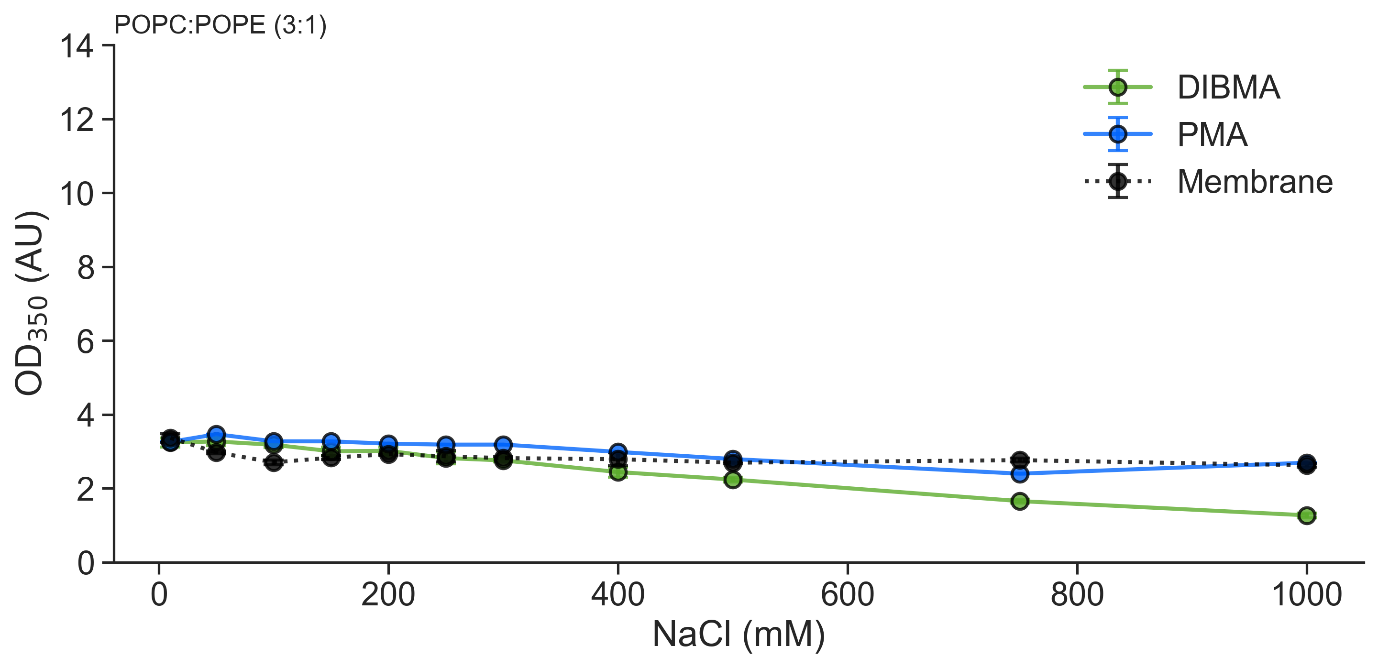


**SI Figure 5** – OD_350_ of NaCl screening. Absorbance at 350 nm for POPC:POPE (3:1) membranes, black line shows absorbance of membrane prior to polymer addition (averaged for all polymer samples). Polymers (DIBMA – green, PMA – blue) were added at 1.5*x* membrane weight (*w/w*), to a final concentration of 1.5%, and incubated at 37^o^C for 1 hour with gentle agitation.

# DLS data


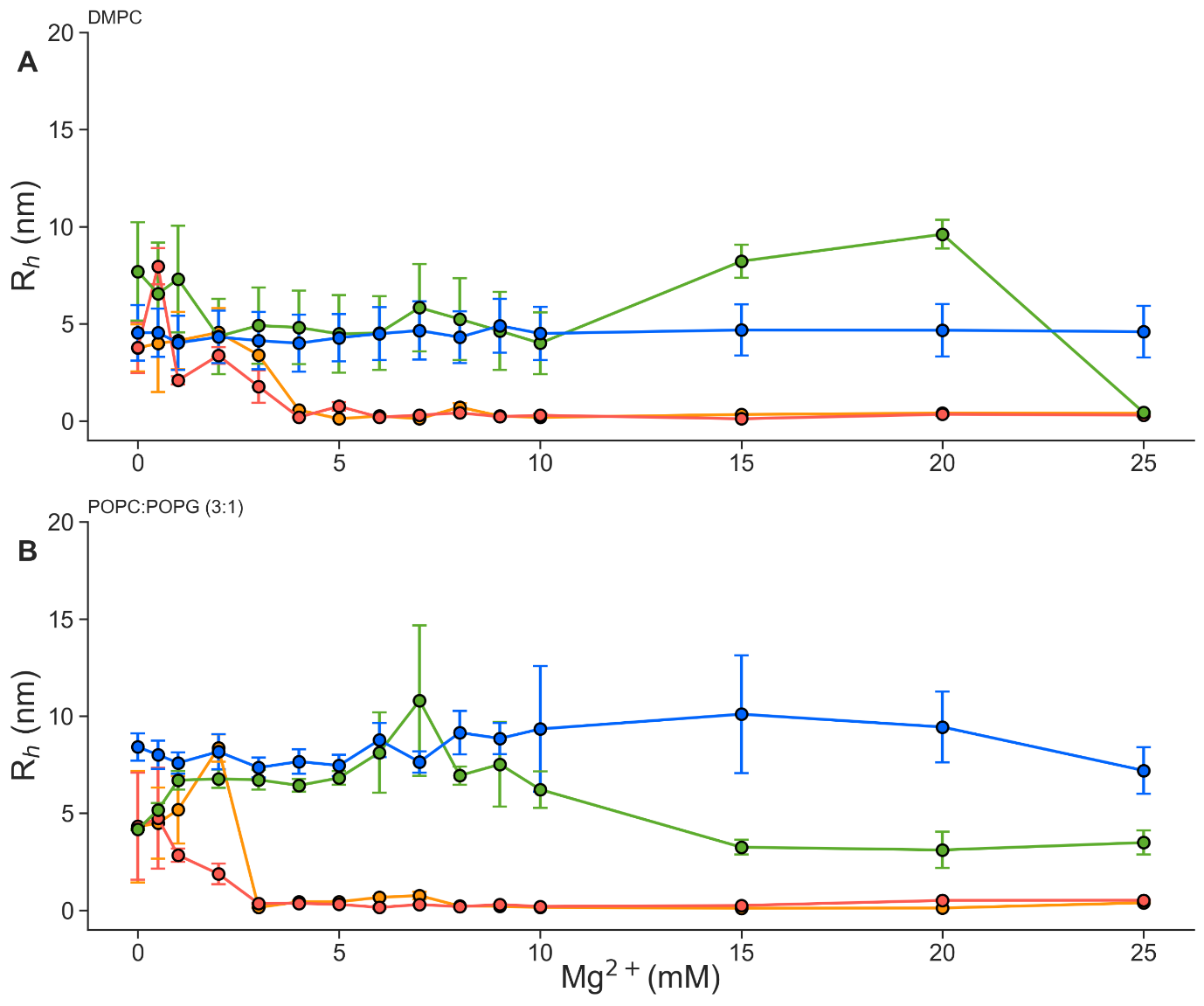


**SI Figure 6** – DLS of Mg^2+^ screening. DLS measurements of polymer-lipid nanoparticles (SMA 3:1 – orange, SMA 2:1 – red, DIBMA – green, PMA – blue) after addition of polymers at 1.5*x* membrane weight (*w/w*), to a final concentration of 1.5%, and incubation at 37^o^C for 1 hour with gentle agitation. Samples were taken directly from OD_350_ measurements and filtered with 0.22 μm to remove aggregates or non-solubilised membrane.


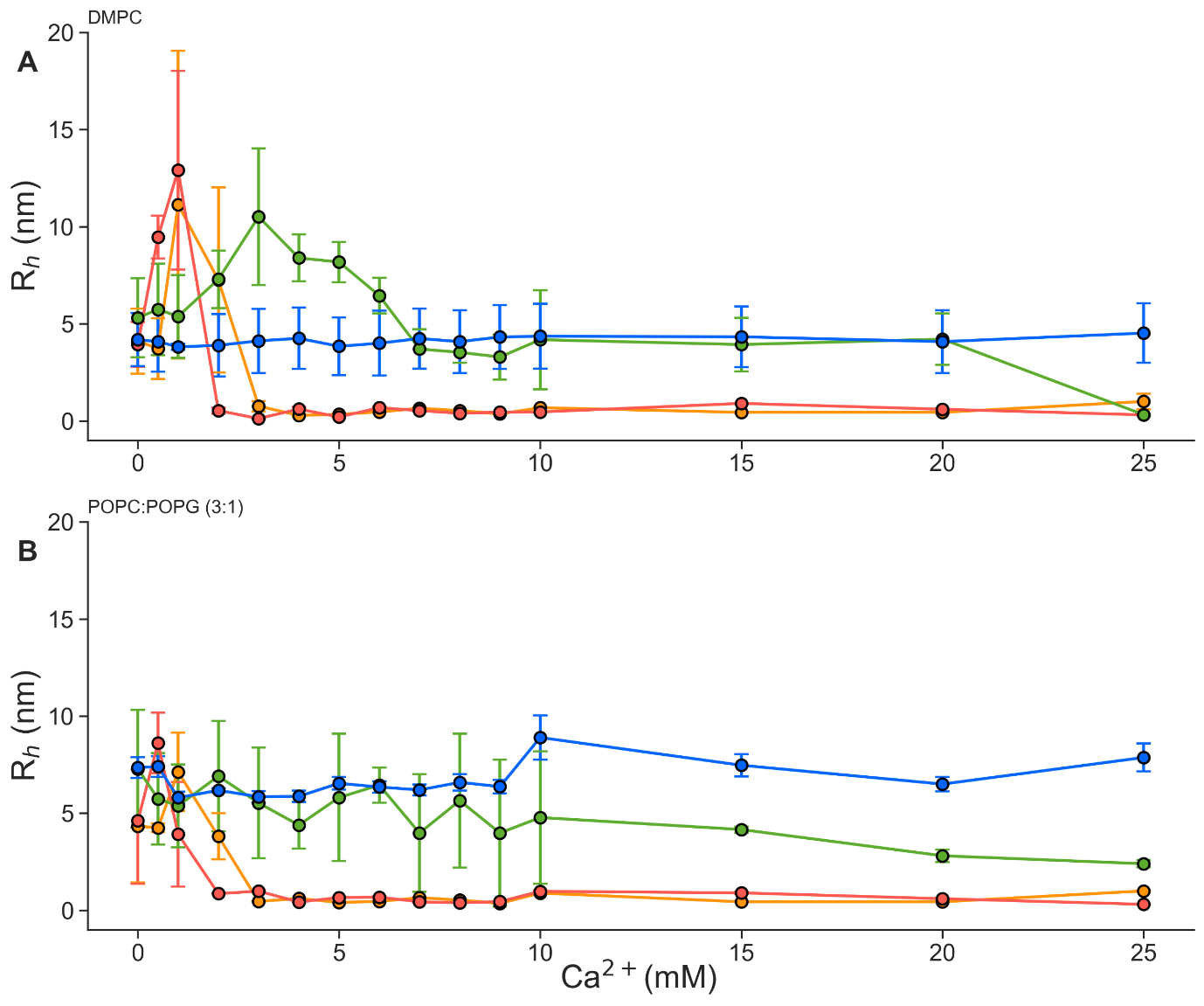


**SI Figure 7** – DLS of Ca^2+^ screening. DLS measurements of polymer-lipid nanoparticles (SMA 3:1 – orange, SMA 2:1 – red, DIBMA – green, PMA – blue) after addition of polymers at 1.5*x* membrane weight (*w/w*), to a final concentration of 1.5%, and incubation at 37^o^C for 1 hour with gentle agitation. Samples were taken directly from OD_350_ measurements and filtered with 0.22 μm to remove aggregates or non-solubilised membrane.

**cw-EPR spectra**


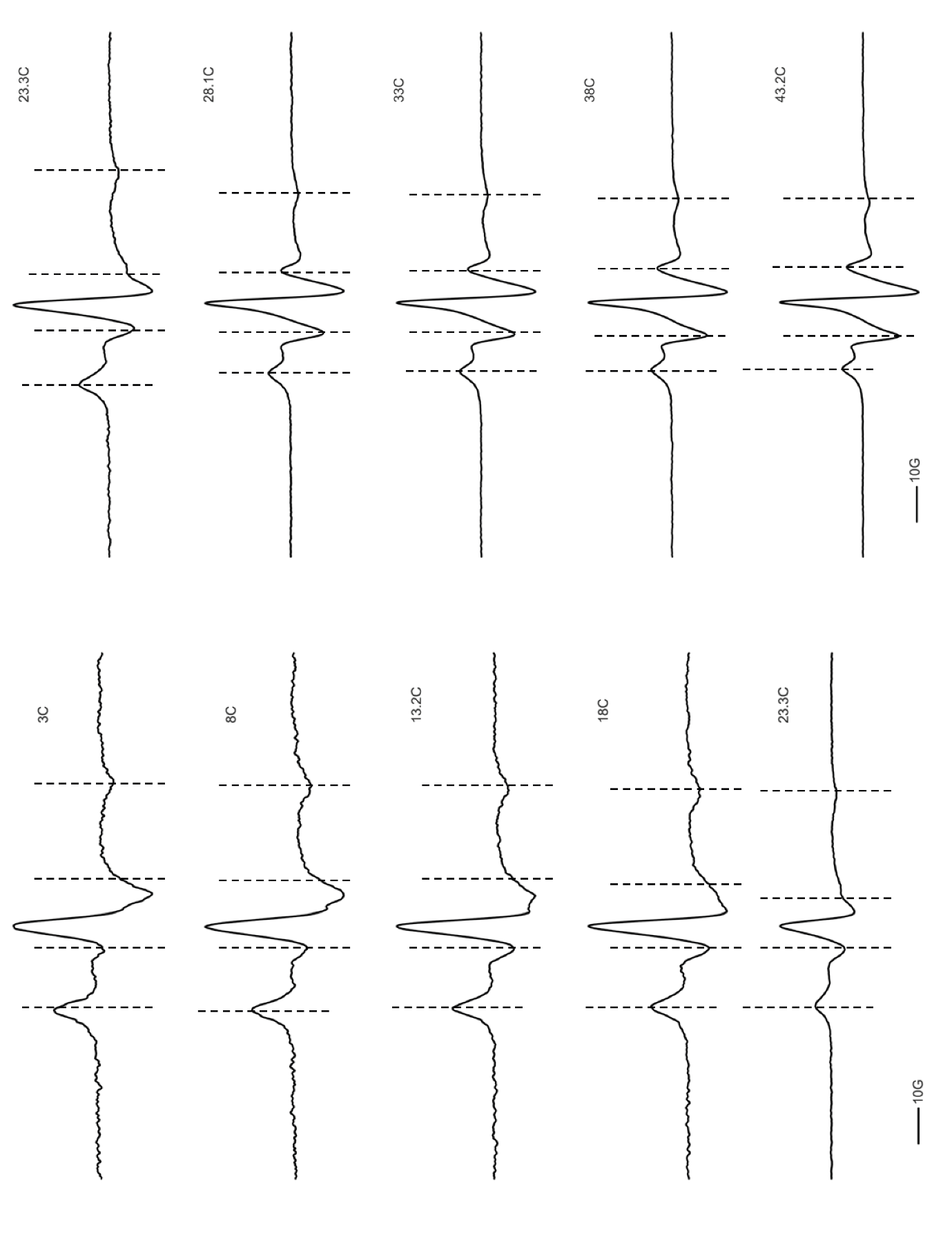


**SI Figure 8** Annotated EPR spectra of 1% (molar) 5-PCSL label within a 400 nm DMPC LUV. Dashed lines show A⊥ (outer pair) and A‖ (inner pair) measuring points. Temperature of measurement for each spectrum is shown in °C (C).


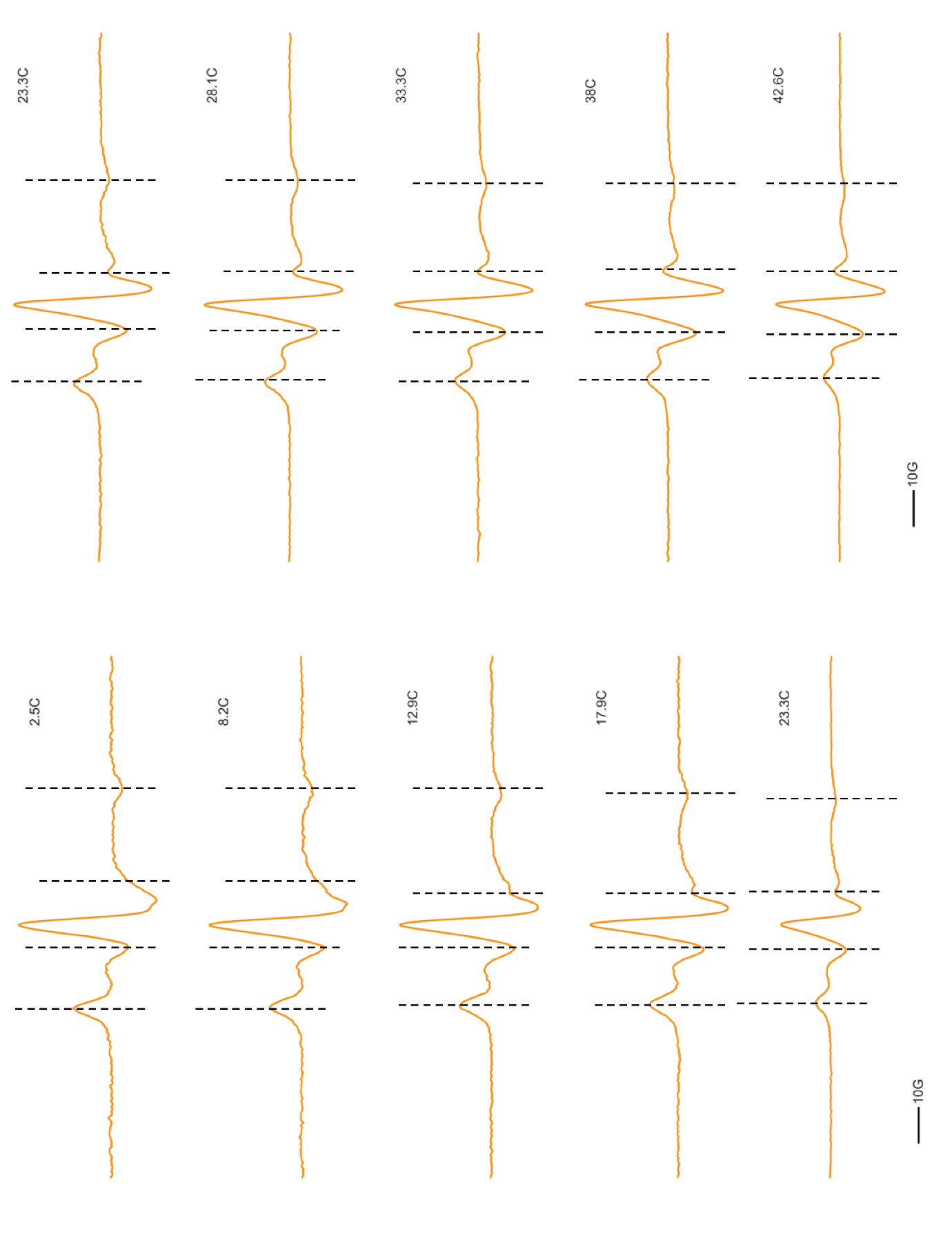


**SI Figure 9** Annotated EPR spectra of 1% (molar) 5-PCSL label within DMPC-SMA 3:1 nanoparticles. Dashed lines show A⊥ (outer pair) and A‖ (inner pair) measuring points. Temperature of measurement for each spectrum is shown in °C (C).


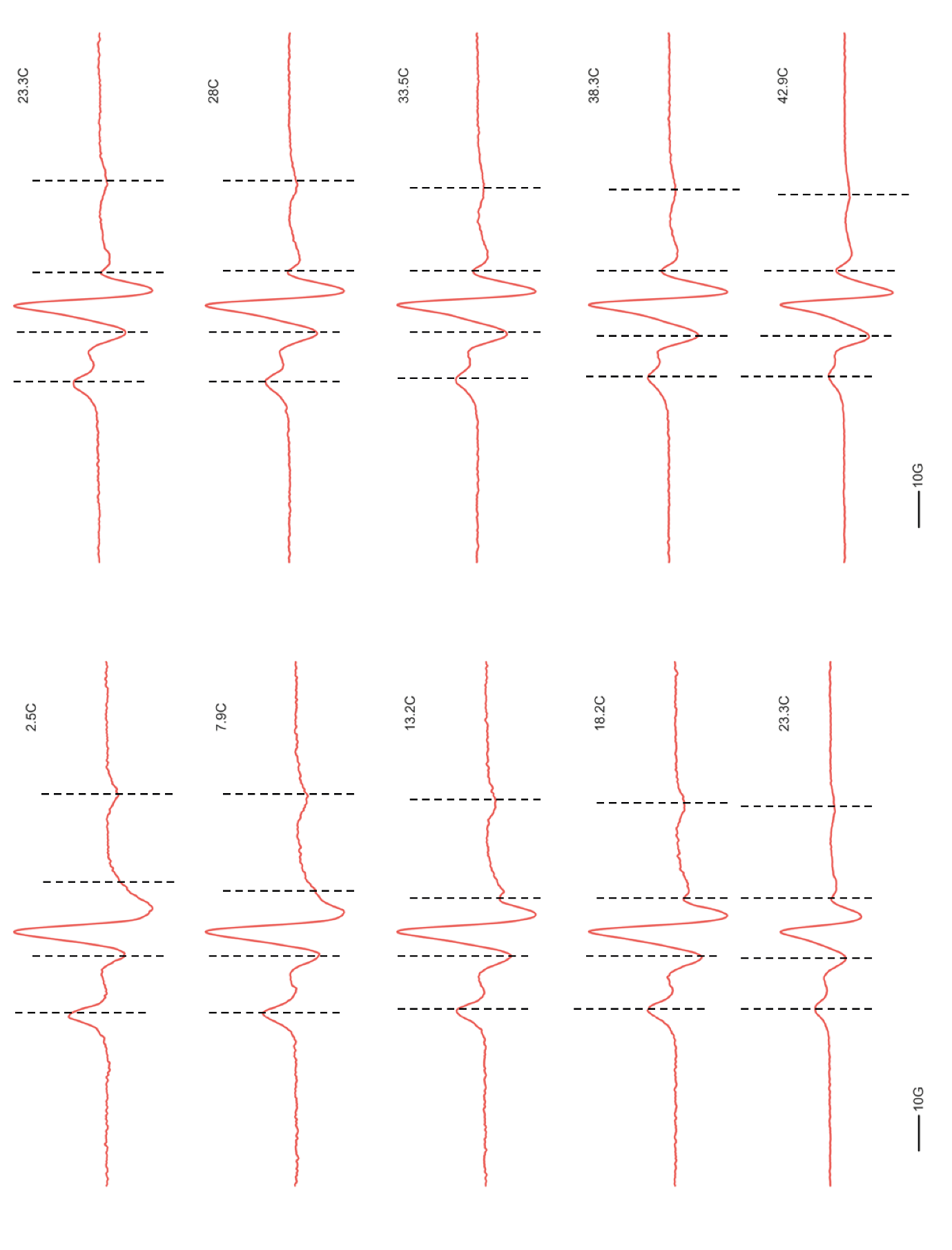


**SI Figure 10** Annotated EPR spectra of 1% (molar) 5-PCSL label within DMPC-SMA 2:1 nanoparticles. Dashed lines show A⊥ (outer pair) and A‖ (inner pair) measuring points. Temperature of measurement for each spectrum is shown in °C (C).


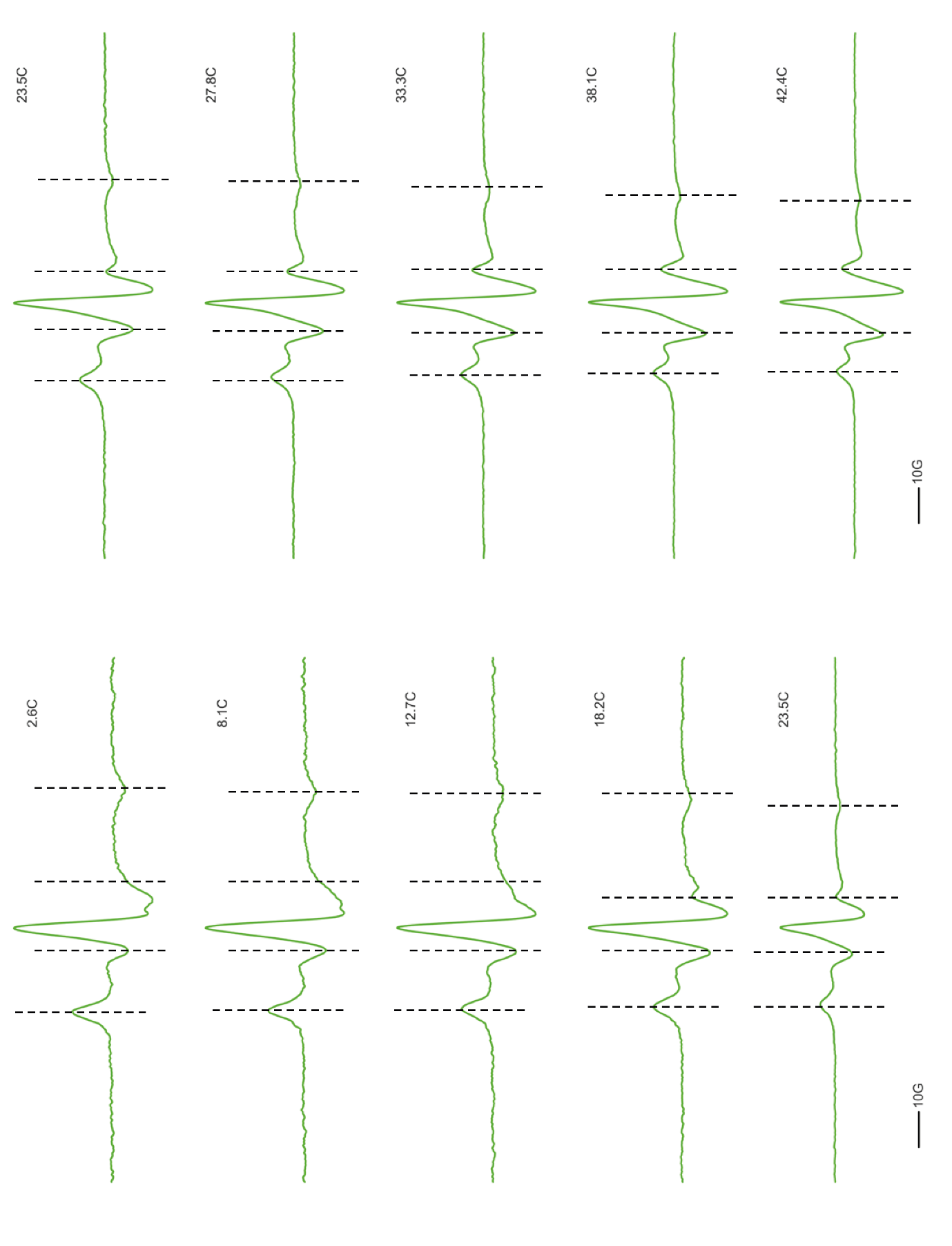


**SI Figure 11** Annotated EPR spectra of 1% (molar) 5-PCSL label within DMPC-DIBMA nanoparticles. Dashed lines show A⊥ (outer pair) and A‖ (inner pair) measuring points. Temperature of measurement for each spectrum is shown in °C (C).


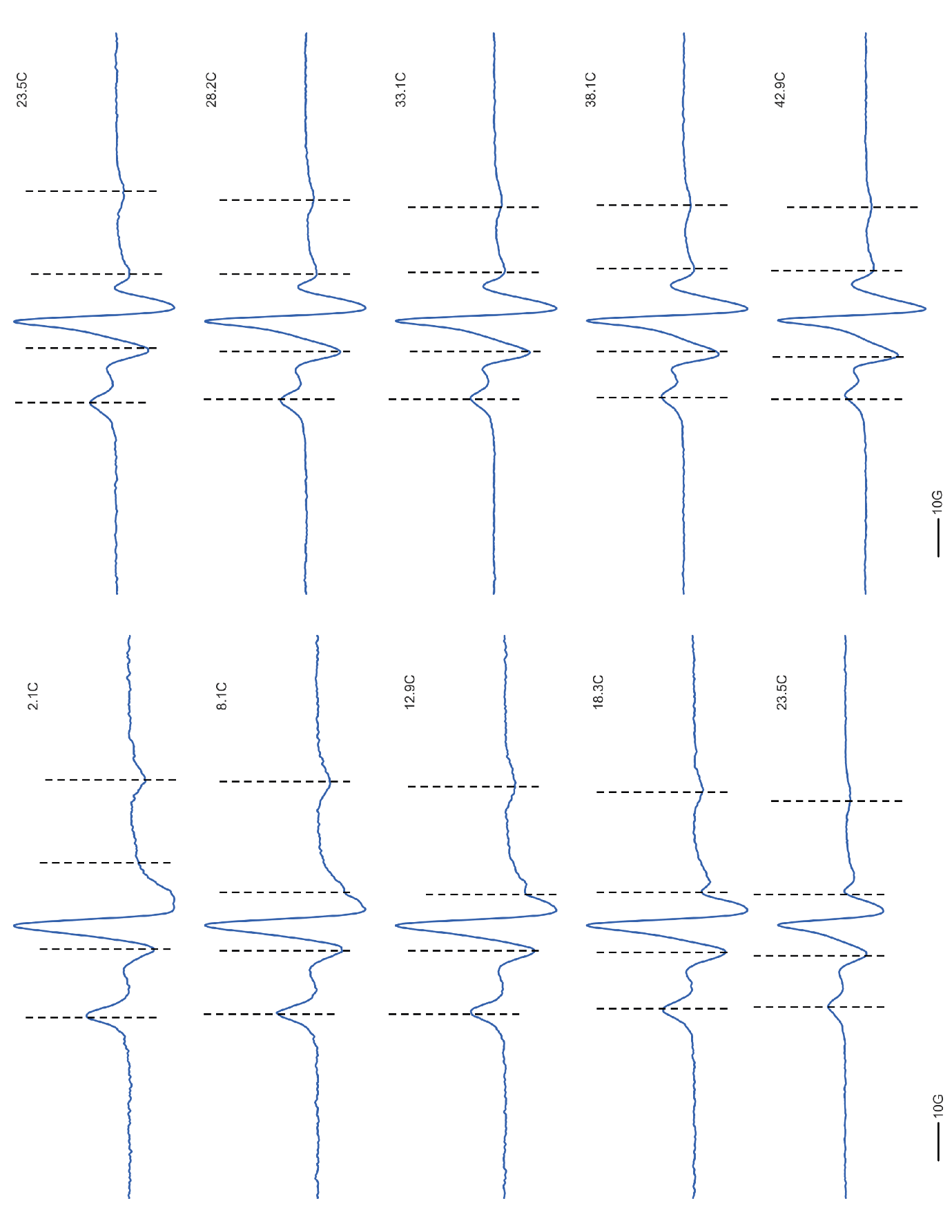


**SI Figure 12** Annotated EPR spectra of 1% (molar) 5-PCSL label within DMPC-PMA nanoparticles. Dashed lines show A⊥ (outer pair) and A‖ (inner pair) measuring points. Temperature of measurement for each spectrum is shown in °C (C).

# TEM nanoparticle measurements

**
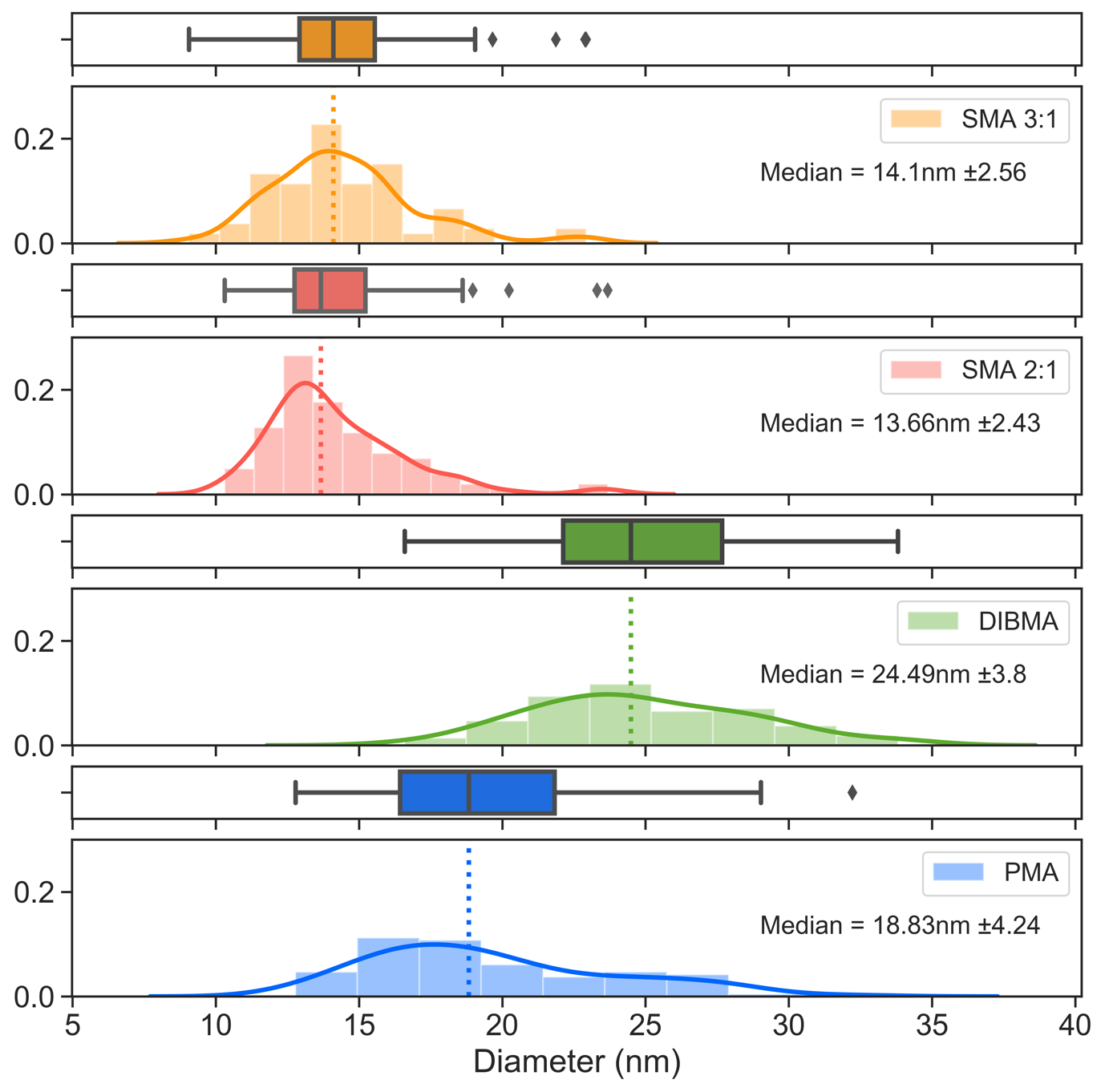
SI Figure 13** – Particle dimension according to TEM. Measurement of major axis per nanoparticle (n=100) for all four polymer types as shown by negative stain TEM. Dotted line represents the median. Black diamonds represent particle dimensions that are statistical outliers.

Particles were surveyed manually using GMS3 software. A total of 100 particles per polymer type were measured along the major axis, with the minor axis measured orthogonally. Median values were calculated through analysis with python, the reported error is the standard deviation. Eccentricity (SI Table 2) was calculated as follows:

$$Eccentricity=\frac{c}{\sqrt{{(b}^{2}+c^{2})}}$$

Minor

Major

$$Eccentricity=\frac{c}{a}$$

c

b

a

**SI Table 2 –** Particle dimensions according to negative stain TEM (average of 100 particles).

| Polymer | Major Axis (nm) | Std. Dev. | Minor Axis (nm) | Std. Dev. | Eccentricity | Std. Dev. |
| --- | --- | --- | --- | --- | --- | --- |
| SMA 3:1 | 14.10 | 2.56 | 11.94 | 2.06 | 0.65 | 0.06 |
| SMA 2:1 | 13.66 | 2.43 | 11.68 | 2.21 | 0.65 | 0.05 |
| DIBMA | 24.49 | 3.80 | 22.37 | 3.73 | 0.64 | 0.03 |
| PMA | 18.83 | 4.24 | 15.45 | 3.27 | 0.68 | 0.05 |

# Summary of published extraction conditions

**SI Table 3** - Review of published conditions used to extract membrane proteins from different membranes in a detergent free manner, in order of publication date.

| Reference | Protein | Expression System | Polymer | Polymer Concentration | pH | NaCl conc. | Incubation Time | Incubation Temp. | Additional Conditions |
| --- | --- | --- | --- | --- | --- | --- | --- | --- | --- |
| (Orwick-Rydmark et al. 2012) | Bacteriorhodopsin (bR) | *H. salinarum* | SMA 3:1 (Lipodisq) | 2.5% (w/v) | 8 | 300 mM | 1 hr | RT | Solubilised in presence of DMPC LUVs |
| (Sahu et al. 2013) | KCNE1 | *E. coli* | SMA 3:1 (Lipodisq) | 1.25% (w/v) | 6.5 | - | Overnight | 4C | Solubilisation of 3:1 POPC:POPG proteoliposomes |
| (Long et al. 2013) | Complex IV | *S. cerevisiae* | SMA 3:1 (Lipodisq) | 2x excess (w/w) | 8 | 200 mM | 20 mins | 26^o^C | Solubilised pre-purified mitochondria |
| (Swainsbury et al. 2014) | Reaction Center (RCs) | *Rba. sphaeroides* | SMA 2:1 (Polyscience) | 1.5% (w/v) | 8 | 100 mM | 1 hr | RT | - |
| (Gulati et al. 2014) | ABCB1, ABCC1, ABCC4, ABCG2, ABCC7 | *Trichoplusia ni* | SMA 2:1 (Polyscience) | 2.5% (w/v) | 8 | 150 mM | 30-60 mins | RT | 10% (v/v) glycerol) |
| (Paulin et al. 2014) | PBP2/PBP2a | *S. aureus* | SMA 2:1 (synthesised in-house) | 2.5% (w/v) | 7.6 | 145 mM | 1 hr | 37^o^C | 20% sucrose (w/v), solubilised pre-purified membranes |
| (Dörr et al. 2014) | KcsA | *E. coli* | SMA 2:1 (Polyscience) | 3% (w/v) | 8 | 300 mM | Overnight | RT | Solubilised pre-disrupted membranes |
| (Jamshad et al. 2015) | hA2AR | *P. pastoris* | SMA 2:1 (Polyscience) | 2.5% (w/v) | 8 | 500 mM | 20 hrs | 25^o^C | 10% glycerol |
| (Jamshad et al. 2015) | hA2AR | *HEK293T cells* | SMA 2:1 (Polyscience) | 2% (w/v) | 7.4 | - | 1 hr | 37^o^C | 1mM magnesium acetate, 1mM EGTA |
| (Bell et al. 2015) | LHC (II) | *S. oleracea* | SMA 3:1 (Polyscience) | 3% (w/v) | 7.6 | - | 20 mins | 4^o^C | 5mM MgCl2, 0.3M sorbitol, solubilised pre-purified thylakoids |
| (Postis et al. 2015) | AcrB | *E. coli* | SMA 2:1 (Polyscience) | 2.5% (w/v) | 8 | 500 mM | 2 hrs | RT | 10% glycerol |
| (Logez et al. 2016) | MT1R | *CHO-K1 cells* | SMA 2:1 (Malvern) | 2.5% (w/v) | 8 | - | * | 4^o^C | Comparable results obtained with SMA 3:1 (LQ) |
| (Logez et al. 2016) | GHS-R1a | *CHO-K1 cells* | SMA 2:1 (Malvern) | 2.5% (w/v) | 8 | - | * | 26^o^C | Comparable results obtained with SMA 3:1 (LQ). |
| (Prabudiansyah et al. 2015) | SecYEG | *E. coli* | SMA 3:1 (Malvern) | 3% (w/v) | 8 | 200 mM | 30 mins | 25^o^C | - |
| (Laursen et al. 2016) | P450 Oxidoreductase (POR) | *S. bicolor* | SMA 2:1 (Polyscience) | 2.5% (w/v) | 7.9 | - | Overnight | 4^o^C | Solubilised microsomal membranes |
| (Komar et al. 2016) | SecYEG super-complex | *E. coli* | SMA 2:1 (Polyscience) | 2.5% (w/v) | 8 | 130 mM | 2 hrs | 21^o^C | 10% glycerol |
| (Voskoboynikova et al. 2017) | NpSRII | *E. coli* | SMA 3:1 (Polyscience) | 2.5% (w/v) | 8 | 150 mM | 1hr then 16 hrs | 4^o^C then RT | Solubilised pre-purified proteoliposomes |
| (Voskoboynikova et al. 2017) | NpSRII/NpHtrII complex | *E. coli* | SMA 3:1 (Polyscience) | 2.5% (w/v) | 8 | 150 mM | 1hr then 16 hrs | 4^o^C then RT | Solubilised pre-purified proteoliposomes |
| (Broecker et al. 2017) | HwBR | *E. coli* | SMA 3:1 (Polyscience) | 2.5% (w/v) | 8 | 300 mM | Overnight | 4^o^C | Additional DMPC during solubilisation (1.5% w/v) |
| (Rehan et al. 2017) | hENT1 | *Sf9 cells* | SMA 2:1 (Polyscience) | 0.25% (w/v) | 8 | 500 mM | 20 hrs | 4^o^C | Solubilised in presence of 0.2% cholesteryl hemisuccinate CHS (w/v) and 10% glycerol |
| (Bersch et al. 2017) | CzcD | *E. coli* | SMA 2:1 (Polyscience) | 0.5% (w/v) | 8.5 | 250 mM | 12 hrs | RT | In 100% D_2_O and 2mM TCEP |
| (Bersch et al. 2017) | CzcD | *E. coli* | SMA 3:1 (Polyscience) | 0.3% (w/v) | 8.5 | 250 mM | 12 hrs | RT | In 100% D_2_O and 2mM TCEP |
| (Bos et al. 2017) | LHC PS(I) | *S. oleracea* | SMA 2:1 (Polyscience) | 3% (w/v) | 7.6 | - | 20 mins | 4^o^C | 5mM MgCl_2_, 0.3M sorbitol, solubilised in absence of light, from pre-purified thylakoid membranes |
| (Reading et al. 2017) | GlpG | *E. coli* | SMA 2:1 (Polyscience) | 2.5% (w/v) | 7.4 | 500 mM | 2 hrs | RT | 10% glycerol |
| (Swainsbury et al. 2018) | Cytochrome bc1 complex (cytbc1) | *Rba. sphaeroides* | SMA 2:1 (Polyscience) | 2.5% (w/v) | 8 | 200 mM | 1 hr | RT | - |
| (Luna et al. 2018) | Human cannabinoid receptor 1 (CB1) | *E. coli* | SMA 2:1 (Polyscience) | 2.5% (w/v) | 8 | 500 mM | Overnight | 4^o^C | - |
| (Damian et al. 2018) | GHSR:D2R heterodimer | *E. coli,*  *P. pastoris* | SMA 2:1 (Malvern) | Lipid:Polymer ratio of 0.1 | 8 | 200 mM | - | 25^o^C | Solubilised from proteoliposomes |
| (Sun et al. 2018) | Alternative complex III (ACIII) | *F. johnsoniae* | SMA 3:1 (Polyscience) | 1% (w/v) | 8 | 300 mM | 1 hr | RT | - |
| (Teo et al. 2019) | ZipA | *E. coli* | SMI | 5% (w/v) | 5 | 200 mM | 2 hrs | RT | Also performed at pH 7, with similar results |
| (Liu et al. 2018) | MraY | *E. coli* | SMA 3:1 (Polyscience) | 3% (w/v) | 7.6 | 150 mM | 1.5 hrs then Overnight | 25^o^C then 4^o^C | Polymer added before cell disruption |
| (Schmidt et al. 2019) | AqpZ | *E. coli* | SMA 3:1 (Polyscience) | 3x excess (w/w) | 8 | 150 mM | Overnight | 4^o^C | - |
| (Barniol-Xicota and Verhelst 2018) | GlpG | *E. coli* | SMA 3:1, SMA 2:1, DIBMA (Polyscience) | 2.5% (w/v) | 8 | 500 mM | 3 hrs | 37^o^C | - |
| (Hellwig et al. 2018) | KtrB | *E. coli* | SMA 3:1 (Polyscience) | 2.5% (w/v) | 8 | 600 mM | 12 hrs | RT | 600mM salt as 420mM NaCl and 180mM KCl |
| (Karlova et al. 2019) | hKCNQ1 | *CHO cells* | SMA 3:1 (Polyscience) | 2.5% (w/v) | 8 | 150 mM | 30 mins | 4^o^C | Sonication during solubilisation, in presence of 2mM DTT |
| (Teo et al. 2019) | FtsA and PgpB | *E. coli* | SMA 2:1 (Polyscience) | 2.5% (w/v) | 8 | 300 mM | ‘Until clear’ | 4^o^C | - |
| (Prakash et al. 2019) | G12-KRAS | *BHK cells* | SMA 3:1 (Malvern) | 2% (w/v) | 7.4 | 137 mM | 1 hr | RT | - |
| (Hardy et al. 2019) | MRP4 | *Sf9 cells* | SMA 2:1 (Polyscience) | 2.5% (w/v) | 7.5 | - | 2 hrs | 4^o^C | 250mM sucrose |
| (D. Dutta, M. Esmaili, M. Overduin 2019) | SOS1 | *P. pastoris* | SMA 2:1 (Polyscience) | 2.5% (w/v) | 8 | 200 mM | 3 hrs | RT | 10% glycerol |
| (Nakatani et al. 2020) | NDH-2a | *E. coli* | SMA 3:1 (Polyscience) | 2.5% (*w/v*) | 8 | 500 mM | 2 hrs | RT | 10% glycerol |
| (Routledge et al. 2020) | A­_2A_R | *P. pastoris* | SMA 3:1 (Polyscience) | 2.5% (*w/v*) | 7.5 | 200 mM | 1 hr | RT | - |
| (Tascón et al. 2020) | KimA | *E. coli* | SMA 2:1 (Polyscience) | 2% (*w/v*) | 8 | 400 mM | Overnight | 4^o^C | 100mM KCl |
| (Horsey et al. 2020) | ABCG2 | *HEK293T* | SMA 2:1 (Polyscience) | 2.5% (*w/v*) | 8 | 150 mM | 1 hr | RT | - |
| (Bada Juarez et al. 2020) | Human dopamine receptor (D1) | *HEK293f* | SMA 3:1 (Malvern) | 1.5x (*w/w*) | 8 | 200 mM | 8 hrs | 4^o^C | - |
| (Hesketh et al. 2020) | AcrB | *E. coli* | SMA 2:1 (Polyscience) | 2.5% (*w/v*) | 8 | 500 mM | 2 hrs | RT | 10% glycerol |
| (Ganapathy et al. 2020) | Green proteorhodopsin (PR) and Gloebacter violaceusrhodopsin (GR) | *E. coli* | SMA 2:1 (Polyscience) | 5% (*w/v*) | 8 | 600 mM | 2-3 days | RT | - |
| (Desrames et al. 2020) | RBC complexes | *H. sapiens* | SMA 3:1 (Malvern) | 2.5% (*w/v*) | 8 | 150 mM | 1 hr | RT | Further incubation overnight at 4^o^C |
| (Swiecicki et al. 2020) | PglC and PglA | *E. coli* | SMA 3:1, SMA 2:1, SMA 1.2:1 (Polyscience) | 2.5% (*w/v*) | 8 | 150 mM | 2 hrs | RT | 10mM imidazole |
| (Gakhar et al. 2020) | bR | *H. salinarium* | SMA 3:1 (Polyscience) | 1.5% (*w/v*) | 7.4 | 100 mM | Unknown | RT | From purple membrane, doped with DMPC LUVs |
| (Cherepanov et al. 2020) | Cyanobacterial photosystem I (PSI) | *T. elongatus* | SMA 1.4:1 (Polyscience) | 1.7% (*w/v*) | 9.5 | 125 mM | 3 hrs | 40^o^C | KCl instead of NaCl |
| (Flegler et al. 2020) | Yna1 | *E. coli* | DIBMA (Polyscience) | 2.5% (*w/v*) | 7.5 | 300 mM | 20 hrs | RT | - |
| (Ueta et al. 2020) | RxR and HsSRI | *E. coli* | SMA 2:1 (Polyscience) | 5% (*w/v*) | 8.5 | 500 mM | 2 hrs | RT | HsSRI was solubilised at 4^o^C |
| (Bernhard and Laube 2020) | GlyR | *HEK293 and X. laevis* | SMA 3:1 (Malvern) | 2% (*w/v*) | 8 | 150 mM | 1 hr | RT | 10% glycerol |
| (Ayub et al. 2020) | CD81 | *P. pastoris* | SMA 2:1 (Polyscience) | 2.5% (*w/v*) | 8 | 150 mM | 1 hr | RT | Also solubilised with 20mM HEPES, 200mM NaCl and 10% glycerol (pH 8) |
| (Johnson et al. 2020) | G288D AcrB | *E. coli* | SMA 2:1 (Polyscience) | 2.5% (*w/v*) | 8 | 500 mM | 2 hrs | RT | - |
| (Lavington and Watts 2021) | NTSR1 | *Sf9 cells* | PMA | 10% (*w/v*) | 7.4 | 500 mM | 1hr | 26^o^C | - |
| (Yu et al. 2021) | GlyR | *Sf9 cells* | SMA 2:1 (Polyscience) | 0.5% (*w/v*) | 7.6 | 150 mM | 1 hr | 4^o^C | - |
| (Voskoboynikova et al. 2021) | Wsc1 | *S. cerevisiae* | SMA 2:1 (Polyscience) | 2.5x (*w/w*) to cell mass | 8 | 150 mM | 30 mins | RT | Further incubation at 4^o^C for 16 hours |
| (Krajewska and Koprowski 2021) | hROMK | *E. coli* | SMA 3:1, SMA 2.3:1, SMA 1.2:1, DIBMA (Polyscience) | 2.5% (*w/v*) | 7.4 | 200 mM | Overnight | 25^o^C | KCl instead of NaCl |
| (Olerinyova et al. 2021) | KcsA | *E. coli* | SMA 3:1 | 1% (*w/v*) | 8 | 300 mM | Overnight | 4^o^C | 15mM KCl, 10mM imidazole |
| (Dilworth et al. 2021) | hSERT | *P. pastoris* | DIBMA,  SMA 3:1 (Polyscience) | 2.5% (*w/v*) | 7.4 | 150 mM | 3 hrs or 16 hrs | RT | 10% glycerol |
| (Patel et al. 2022) | BK | *HEK293* | SMA 2:1 (Polyscience) | 2.5% | 8 | 150mM | 1 hr | RT | - |

# SI References

Ayub H, Clare M, Milic I, et al (2020) CD81 extracted in SMALP nanodiscs comprises two distinct protein populations within a lipid environment enriched with negatively charged headgroups. Biochim Biophys Acta Biomembr 1862:183419. https://doi.org/10.1016/j.bbamem.2020.183419

Bada Juarez JF, Muñoz-García JC, Inácio dos Reis R, et al (2020) Detergent-free extraction of a functional low-expressing GPCR from a human cell line. Biochim Biophys Acta Biomembr 1862:183152. https://doi.org/10.1016/j.bbamem.2019.183152

Barniol-Xicota M, Verhelst SHL (2018) Stable and Functional Rhomboid Proteases in Lipid Nanodiscs by Using Diisobutylene/Maleic Acid Copolymers. J Am Chem Soc 140:14557–14561. https://doi.org/10.1021/jacs.8b08441

Bell AJ, Frankel LK, Bricker TM (2015) High yield non-detergent isolation of photosystem I-light-harvesting chlorophyll II membranes from spinach thylakoids: Implications for the organization of the PS I antennae in higher plants. Journal of Biological Chemistry 290:18429–18437. https://doi.org/10.1074/jbc.M115.663872

Bernhard M, Laube B (2020) Thermophoretic analysis of ligand-specific conformational states of the inhibitory glycine receptor embedded in copolymer nanodiscs. Sci Rep 10:1–11. https://doi.org/10.1038/s41598-020-73157-2

Bersch B, Dörr JM, Hessel A, et al (2017) Proton-Detected Solid-State NMR Spectroscopy of a Zinc Diffusion Facilitator Protein in Native Nanodiscs. Angewandte Chemie - International Edition 56:2508–2512. https://doi.org/10.1002/anie.201610441

Bos I, Bland KM, Tian L, et al (2017) Multiple LHCII antennae can transfer energy efficiently to a single Photosystem I. Biochim Biophys Acta Bioenerg 1858:371–378. https://doi.org/10.1016/j.bbabio.2017.02.012

Broecker J, Eger BT, Ernst OP (2017) Crystallogenesis of Membrane Proteins Mediated by Polymer-Bounded Lipid Nanodiscs. Structure 25:384–392. https://doi.org/10.1016/j.str.2016.12.004

Cherepanov DA, Brady NG, Shelaev I V., et al (2020) PSI-SMALP, a Detergent-free Cyanobacterial Photosystem I, Reveals Faster Femtosecond Photochemistry. Biophys J 118:337–351. https://doi.org/10.1016/j.bpj.2019.11.3391

D. Dutta, M. Esmaili, M. Overduin LF (2019) Expression and Detergent Free Purification and Reconstitution of the Plant Plasma Membrane Na+/H+ Antiporter SOS1 Overexpressed in Pichia pastoris. BBA - Biomembranes 105:72–80. https://doi.org/10.1016/j.neubiorev.2019.07.019

Damian M, Pons V, Renault P, et al (2018) GHSR-D2R heteromerization modulates dopamine signaling through an effect on G protein conformation. Proc Natl Acad Sci U S A 115:4501–4506. https://doi.org/10.1073/pnas.1712725115

Desrames A, Genetet S, Delcourt MP, et al (2020) Detergent-free isolation of native red blood cell membrane complexes. Biochim Biophys Acta Biomembr 1862:183126. https://doi.org/10.1016/j.bbamem.2019.183126

Dilworth M V., Findlay HE, Booth PJ (2021) Detergent-free purification and reconstitution of functional human serotonin transporter (SERT) using diisobutylene maleic acid (DIBMA) copolymer. Biochimica et Biophysica Acta (BBA) - Biomembranes 1863:183602. https://doi.org/10.1016/j.bbamem.2021.183602

Dörr JM, Koorengevel MC, Schäfer M, et al (2014) Detergent-free isolation, characterization, and functional reconstitution of a tetrameric K+ channel: The power of native nanodiscs. Proc Natl Acad Sci U S A 111:18607–18612. https://doi.org/10.1073/pnas.1416205112

Flegler VJ, Rasmussen A, Rao S, et al (2020) The MscS-like channel YnaI has a gating mechanism based on flexible pore helices. Proc Natl Acad Sci U S A 117:28754–28762. https://doi.org/10.1073/pnas.2005641117

Gakhar S, Risbud SH, Longo ML (2020) Structure retention of silica gel-encapsulated bacteriorhodopsin in purple membrane and in lipid nanodiscs. Colloids Surf B Biointerfaces 186:110680. https://doi.org/10.1016/j.colsurfb.2019.110680

Ganapathy S, Opdam L, Hontani Y, et al (2020) Membrane matters: The impact of a nanodisc-bilayer or a detergent microenvironment on the properties of two eubacterial rhodopsins. Biochim Biophys Acta Biomembr 1862:183113. https://doi.org/10.1016/j.bbamem.2019.183113

Gulati S, Jamshad M, Knowles TJ, et al (2014) Detergent-free purification of ABC (ATP-binding-cassette) transporters. Biochemical Journal 461:269–278. https://doi.org/10.1042/BJ20131477

Hardy D, Bill RM, Rothnie AJ, Jawhari A (2019) Stabilization of Human Multidrug Resistance Protein 4 (MRP4/ABCC4) Using Novel Solubilization Agents. SLAS DISCOVERY: Advancing Life Sciences R&D 00:247255521986707. https://doi.org/10.1177/2472555219867074

Hellwig N, Peetz O, Ahdash Z, et al (2018) Native mass spectrometry goes more native: Investigation of membrane protein complexes directly from SMALPs. Chemical Communications 54:13702–13705. https://doi.org/10.1039/c8cc06284f

Hesketh SJ, Klebl DP, Higgins AJ, et al (2020) Styrene maleic-acid lipid particles (SMALPs) into detergent or amphipols: An exchange protocol for membrane protein characterisation. Biochim Biophys Acta Biomembr 1862:183192. https://doi.org/10.1016/j.bbamem.2020.183192

Horsey AJ, Briggs DA, Holliday ND, et al (2020) Application of fluorescence correlation spectroscopy to study substrate binding in styrene maleic acid lipid copolymer encapsulated ABCG2. Biochim Biophys Acta Biomembr 1862:183218. https://doi.org/10.1016/j.bbamem.2020.183218

Jamshad M, Charlton J, Lin YP, et al (2015) G-protein coupled receptor solubilization and purification for biophysical analysis and functional studies, in the total absence of detergent. Biosci Rep 35:1–10. https://doi.org/10.1042/BSR20140171

Johnson RM, Fais C, Parmar M, et al (2020) Cryo-EM structure and molecular dynamics analysis of the fluoroquinolone resistant mutant of the acrb transporter from salmonella. Microorganisms 8:1–21. https://doi.org/10.3390/microorganisms8060943

Karlova MG, Voskoboynikova N, Gluhov GS, et al (2019) Detergent-free solubilization of human Kv channels expressed in mammalian cells. Chem Phys Lipids 219:50–57. https://doi.org/10.1016/j.chemphyslip.2019.01.013

Komar J, Alvira S, Schulze RJ, et al (2016) Membrane protein insertion and assembly by the bacterial holo-Translocon SecYEG-SecDF-YajC-YidC. Biochemical Journal 473:3341–32354. https://doi.org/10.1042/BCJ20160545

Krajewska M, Koprowski P (2021) Solubilization, purification, and functional reconstitution of human ROMK potassium channel in copolymer styrene-maleic acid (SMA) nanodiscs. Biochim Biophys Acta Biomembr 1863:183555. https://doi.org/10.1016/j.bbamem.2021.183555

Laursen T, Borch J, Knudsen C, et al (2016) Characterization of a dynamic metabolon producing the defense compound dhurrin in sorghum. Science (1979) 354:890–893. https://doi.org/10.1126/science.aag2347

Lavington S, Watts A (2021) Detergent-free solubilisation &amp; purification of a G protein coupled receptor using a polymethacrylate polymer. Biochimica et Biophysica Acta (BBA) - Biomembranes 1863:183441. https://doi.org/10.1016/j.bbamem.2020.183441

Liu Y, Moura ECCM, Dörr JM, et al (2018) Bacillus subtilis MraY in detergent-free system of nanodiscs wrapped by styrene-maleic acid copolymers. PLoS One 13:1–18. https://doi.org/10.1371/journal.pone.0206692

Logez C, Damian M, Legros C, et al (2016) Detergent-free Isolation of Functional G Protein-Coupled Receptors into Nanometric Lipid Particles. Biochemistry 55:38–48. https://doi.org/10.1021/acs.biochem.5b01040

Long AR, O’Brien CC, Malhotra K, et al (2013) A detergent-free strategy for the reconstitution of active enzyme complexes from native biological membranes into nanoscale discs. BMC Biotechnol 13:41. https://doi.org/10.1186/1472-6750-13-41

Luna VM, Vazir M, Vaish A, et al (2018) Generation of membrane proteins in polymer-based lipoparticles as flow cytometry antigens. Eur Polym J 109:483–488. https://doi.org/10.1016/j.eurpolymj.2018.10.017

Nakatani Y, Shimaki Y, Dutta D, et al (2020) Unprecedented Properties of Phenothiazines Unraveled by a NDH-2 Bioelectrochemical Assay Platform. J Am Chem Soc 142:1311–1320. https://doi.org/10.1021/jacs.9b10254

Olerinyova A, Sonn-Segev A, Gault J, et al (2021) Mass Photometry of Membrane Proteins. Chem 7:224–236. https://doi.org/10.1016/j.chempr.2020.11.011

Orwick-Rydmark M, Lovett JE, Graziadei A, et al (2012) Detergent-free incorporation of a seven-transmembrane receptor protein into nanosized bilayer lipodisq particles for functional and biophysical studies. Nano Lett 12:4687–4692. https://doi.org/10.1021/nl3020395

Patel JH, Pollock NL, Maher J, et al (2022) The function of BK channels extracted and purified within SMALPs. Biochem J 479:1609–1619. https://doi.org/10.1042/BCJ20210628

Paulin S, Jamshad M, Dafforn TR, et al (2014) Surfactant-free purification of membrane protein complexes from bacteria: Application to the staphylococcal penicillin-binding protein complex PBP2/PBP2a. Nanotechnology 25:. https://doi.org/10.1088/0957-4484/25/28/285101

Postis V, Rawson S, Mitchell JK, et al (2015) The use of SMALPs as a novel membrane protein scaffold for structure study by negative stain electron microscopy. Biochim Biophys Acta Biomembr 1848:496–501. https://doi.org/10.1016/j.bbamem.2014.10.018

Prabudiansyah I, Kusters I, Caforio A, Driessen AJM (2015) Characterization of the annular lipid shell of the Sec translocon. Biochim Biophys Acta Biomembr 1848:2050–2056. https://doi.org/10.1016/j.bbamem.2015.06.024

Prakash P, Litwin D, Liang H, et al (2019) Dynamics of Membrane-Bound G12V-KRAS from Simulations and Single-Molecule FRET in Native Nanodiscs. Biophys J 116:179–183. https://doi.org/10.1016/j.bpj.2018.12.011

Reading E, Hall Z, Martens C, et al (2017) Interrogating Membrane Protein Conformational Dynamics within Native Lipid Compositions. Angewandte Chemie - International Edition 56:15654–15657. https://doi.org/10.1002/anie.201709657

Rehan S, Paavilainen VO, Jaakola VP (2017) Functional reconstitution of human equilibrative nucleoside transporter-1 into styrene maleic acid co-polymer lipid particles. Biochim Biophys Acta Biomembr 1859:1059–1065. https://doi.org/10.1016/j.bbamem.2017.02.017

Routledge SJ, Jamshad M, Little HA, et al (2020) Ligand-induced conformational changes in a SMALP-encapsulated GPCR. Biochim Biophys Acta Biomembr 1862:. https://doi.org/10.1016/j.bbamem.2020.183235

Sahu ID, McCarrick RM, Troxel KR, et al (2013) DEER EPR measurements for membrane protein structures via bifunctional spin labels and lipodisq nanoparticles. Biochemistry 52:6627–6632. https://doi.org/10.1021/bi4009984

Schmidt V, Sidore M, Bechara C, et al (2019) The lipid environment of Escherichia coli Aquaporin Z. Biochim Biophys Acta Biomembr 1861:431–440. https://doi.org/10.1016/j.bbamem.2018.10.017

Sun C, Benlekbir S, Venkatakrishnan P, et al (2018) Structure of the alternative complex III in a supercomplex with cytochrome oxidase. Nature 557:123–126. https://doi.org/10.1038/s41586-018-0061-y

Swainsbury DJK, Proctor MS, Hitchcock A, et al (2018) Probing the local lipid environment of the Rhodobacter sphaeroides cytochrome bc1 and Synechocystis sp. PCC 6803 cytochrome b6f complexes with styrene maleic acid. Biochim Biophys Acta Bioenerg 1859:215–225. https://doi.org/10.1016/j.bbabio.2017.12.005

Swainsbury DJK, Scheidelaar S, Van Grondelle R, et al (2014) Bacterial reaction centers purified with styrene maleic acid copolymer retain native membrane functional properties and display enhanced stability. Angewandte Chemie - International Edition 53:11803–11807. https://doi.org/10.1002/anie.201406412

Swiecicki JM, Santana JT, Imperiali B (2020) A Strategic Approach for Fluorescence Imaging of Membrane Proteins in a Native-like Environment. Cell Chem Biol 27:245-251.e3. https://doi.org/10.1016/j.chembiol.2019.11.008

Tascón I, Sousa JS, Corey RA, et al (2020) Structural basis of proton-coupled potassium transport in the KUP family. Nat Commun 11:1–10. https://doi.org/10.1038/s41467-020-14441-7

Teo ACK, Lee SC, Pollock NL, et al (2019) Analysis of SMALP co-extracted phospholipids shows distinct membrane environments for three classes of bacterial membrane protein. Sci Rep 9:1–10. https://doi.org/10.1038/s41598-018-37962-0

Ueta T, Kojima K, Hino T, et al (2020) Applicability of Styrene-Maleic Acid Copolymer for Two Microbial Rhodopsins, RxR and HsSRI. Biophys J 119:1760–1770. https://doi.org/10.1016/j.bpj.2020.09.026

Voskoboynikova N, Karlova M, Kurre R, et al (2021) A Three-Dimensional Model of the Yeast Transmembrane Sensor Wsc1 Obtained by SMA-Based Detergent-Free Purification and Transmission Electron Microscopy. Journal of Fungi 7:118. https://doi.org/10.3390/jof7020118

Voskoboynikova N, Mosslehy W, Colbasevici A, et al (2017) Characterization of an archaeal photoreceptor/transducer complex from: Natronomonas pharaonis assembled within styrene-maleic acid lipid particles. RSC Adv 7:51324–51334. https://doi.org/10.1039/c7ra10756k

Yu J, Zhu H, Lape R, et al (2021) Mechanism of gating and partial agonist action in the glycine receptor. Cell 184:957-968.e21. https://doi.org/10.1016/j.cell.2021.01.026
